# Understanding of the structural chemistry in the uranium oxo-tellurium system under HT/HP conditions

**DOI:** 10.3389/fchem.2023.1152113

**Published:** 2023-03-10

**Authors:** Yucheng Hao, Eike M. Langer, Bin Xiao, Philip Kegler, Xin Cao, Kunhong Hu, Rüdiger-A. Eichel, Shuao Wang, Evgeny V. Alekseev

**Affiliations:** ^1^ School of Energy Materials and Chemical Engineering, Hefei University, Hefei, China; ^2^ Institute of Energy and Climate Research (IEK-6), Forschungszentrum Jülich GmbH, Jülich, Germany; ^3^ Institute of Energy and Climate Research (IEK-9), Forschungszentrum Jülich GmbH, Jülich, Germany; ^4^ Institut für Materialien und Prozesse für Elektrochemische Energiespeicher-und Wandler, RWTH Aachen University, Aachen, Germany; ^5^ State Key Laboratory of Radiation Medicine and Protection, School for Radiological and Interdisciplinary Sciences (RAD-X) and Collaborative Innovation Center of Radiation Medicine of Jiangsu Higher Education Institutions, Soochow University, Suzhou, China

**Keywords:** highpressure, uranium, tellurium, crystals, crystal structure, hightemperature

## Abstract

The study of phase formation in the U-Te-O systems with mono and divalent cations under high-temperature high-pressure (HT/HP) conditions has resulted in four new inorganic compounds: K_2_ [(UO_2_) (Te_2_O_7_)], Mg [(UO_2_) (TeO_3_)_2_], Sr [(UO_2_) (TeO_3_)_2_] and Sr [(UO_2_) (TeO_5_)]. Tellurium occurs as Te^IV^, Te^V^, and Te^VI^ in these phases which demonstrate the high chemical flexibility of the system. Uranium VI) adopts a variety of coordinations, namely, UO_6_ in K_2_ [(UO_2_) (Te_2_O_7_), UO_7_ in Mg [(UO_2_) (TeO_3_)_2_] and Sr [(UO_2_) (TeO_3_)_2_], and UO_8_ in Sr [(UO_2_) (TeO_5_)]. The structure of K_2_ [(UO_2_) (Te_2_O_7_)] is featured with one dimensional (1D) [Te_2_O_7_]^4-^ chains along the *c*-axis. The Te_2_O_7_ chains are further linked by UO_6_ polyhedra, forming the 3D [(UO_2_) (Te_2_O_7_)]^2-^ anionic frameworks. In Mg [(UO_2_) (TeO_3_)_2_], TeO_4_ disphenoids share common corners with each other resulting in infinite 1D chains of [(TeO_3_)_2_]^4-^ propagating along the *a*-axis. These chains link the uranyl bipyramids by edge sharing along two edges of the disphenoids, resulting in the 2D layered structure of [(UO_2_) (Te_2_O_6_)]^2-^. The structure of Sr [(UO_2_) (TeO_3_)_2_] is based on 1D chains of [(UO_2_) (TeO_3_)_2_]_∞_^2−^ propagating into the *c*-axis. These chains are formed by edge-sharing uranyl bipyramids which are additionally fused together by two TeO_4_ disphenoids, which also share two edges. The 3D framework structure of Sr [(UO_2_) (TeO_5_)] is composed of 1D [TeO_5_]^4−^ chains sharing edges with UO_7_ bipyramids. Three tunnels based on 6-Membered rings (MRs) are propagating along [001] [010] and [100] directions. The HT/HP synthetic conditions for the preparation of single crystalline samples and their structural aspects are discussed in this work.

## 1 Introduction

The structural and chemical diversity of oxo-tellurium and uranium bearing phases has attracted researchers in the field of solid state and materials chemistry, especially in regard to the diverse oxidation states and coordination geometries that tellurium and uranium can adopt in oxo-based phases (Christy et al., 2016). In nature, a number of minerals within this family have been reported: cliffordite [(UO_2_) (Te_3_O_7_)] (Brandstatter, 1981), moctezumite (PbUO_2_(TeO_3_)_2_) (Swihart et al., 1993), schmitterite (UO_2_(TeO_3_) (Meunier and Galy, 1973), and more recently markcooperite (Pb_2_(UO_2_)TeO_6_) (Kampf et al., 2010). Besides the attention due to the diverse fundamental aspects of inorganic chemistry, the presence of tellurium within spent nuclear fuel and its corrosive capabilities also makes this thematic relevant for environmental issues (Kleykamp, 1985). Moctezumite and schmitterite, for example, are secondary minerals commonly observed in telluride-bearing ores (Swihart et al., 1993).

The predominant oxidation state of uranium in oxidizing conditions is U^VI^. Hereby, uranium is present as almost linear trans dioxo-cations (UO_2_^2+^, the so called uranyl group) both in solid state as well as in solution. Within the solid state, the typical coordination environment surrounding the dioxo-cations is a bipyramid, in which the uranyl forms the central axis and additional four to six oxygen atoms occupy sites within the equatorial plane, leading to tetragonal, pentagonal or hexagonal bipyramids (Burns et al., 1997; Hao et al., 2016; Hao et al., 2017a; Hao et al., 2017b; Hao et al., 2018; Hao et al., 2020a; Hao et al., 2022).

Tellurium is typically present as Te^IV^ or Te^VI^ and hereby in form of oxo-anions, tellurites and tellurates, respectively. Te^IV^ is known to adopt several coordination environments, for example, pyramidal TeO_3_, disphenoidal TeO_4_ and square pyramidal TeO_5_ (Balraj and Vidyasagar, 1999; Kim et al., 2007a; Kim et al., 2010). A further inter-connection *via* corner-sharing can lead to complex oxo-tellurium polymers (Lindqvist and Moret, 1973; Hafidi et al., 1986; Mao et al., 2008; Lin et al., 2013). In some compounds several coordination environments of Te can be found, for example, NH_4_ *A*Te_4_O_9_⋅2H_2_O (*A* = Rb, Cs) in which all three coordination geometries of Te are present (Kim and Halasyamani, 2008). Additionally, the lone electron pairs in Te^IV^ have a strong influence on the diversity of Te-O coordination environments. The hexavalent Te (Te^VI^) typically adopts trigonal bipyramidal, distorted octahedral or tetrahedral coordination in oxygen phases. A few ditellurates contain mixed valent tellurium, Te^IV^ and Te^VI^, *A*CuTe_2_O_7_ (*A* = Sr, Ba, or Pb) and Ba*M*Te_2_O_7_ (*M* = Mg or Zn) have been previously reported (Yeon et al., 2011; Yeon et al., 2012). Compared to Te^IV^ or Te^VI^, which are more stable at ambient conditions, inorganic Te^V^ bearing phases were scarcely reported (Lindqvist and Moret, 1973; Hafidi et al., 1986; Balraj and Vidyasagar, 1999; Kim et al., 2007a; Mao et al., 2008; Kim et al., 2010; Lin et al., 2013).

The different structural units have a pronounced influence on the dimensionality of the resulting phases. In the presence of hexavalent uranium with uranyl groups, typically two-dimensional structures dominate (Burns et al., 1996; Burns et al., 1997). This is a direct consequence of oxo-anions typically only being able to condense perpendicular to the terminal uranyl group of the uranyl polyhedra (Burns et al., 1997; Hao et al., 2016; Hao et al., 2017a; Hao et al., 2017b; Hao et al., 2018; Hao et al., 2020; Hao et al., 2022). However, less than half of the currently known phases in uranyl oxo-tellurium system (15 of 32 found in the ICSD (Bergerhoff et al., 1987)), crystallize as two-dimensional structures. In this atypical formation of many one- and three-dimensional structures, the presence of the stereochemically active lone pair, plays a central role (Almond and Albrecht-Schmitt, 2002; Xiao et al., 2016a).

From a materials science point-of-view, studies on tellurium-based phases have been focused on the synthesis of non-centrosymmetric phases (NCS). These phases are of interest for potential applications in the fields of second harmonic generation (SHG) as well as ferro- and piezo-and pyroelectricity (Almond and Albrecht-Schmitt, 2002; Xiao et al., 2016a). This acentric behavior is also addressed by the presence of the aforementioned stereochemically active lone pairs present in Te^IV^. This has resulted in rich results of NCS crystal structures in recent years (Chi et al., 2006; Kim et al., 2014). However, the presence of acentric tellurite groups does not necessarily need to result in NCS phases. The acentric units can order themselves to counteract a potential global NCS structure (Chi et al., 2006; Kim et al., 2007b; Kim et al., 2014).

We have recently systematically studied the *A*-U-Te-O (*A* = alkali and alkaline Earth metal) system under extreme conditions (HT/HP). Our goal is to further understand the different chemical behavior of actinide-tellurium oxo-phases from extreme conditions compared to conventional ones and to develop a methodology of how these phases crystallize under the extreme environment. As a result of this study, a series of quaternary oxide tellurium materials, K_2_ [(UO_2_) (Te_2_O_7_)], Mg [(UO_2_) (TeO_3_)_2_], Sr [(UO_2_) (TeO_3_)_2_] and Sr [(UO_2_) (TeO_5_)] have been prepared by the HT/HP solid state reaction method. In which, K_2_ [(UO_2_) (Te_2_O_7_)] is a very rare example of a Te^V^ bearing phase. The detailed HT/HP synthetic routes, high-temperature and high-pressure behavior, and topology of the structures are discussed.

## 2 Experimental section

***Caution!*** *The UO*_*2*_(*NO*_*3*_)_*2*_ *⋅6H*_*2*_ *O used in this work contained natural uranium; nevertheless the standard precautions for handling radioactive materials must be followed. The γ-UO*_*3*_ *was formed simply by heating the uranyl nitrate and analyzing it with powder XRD* (Engmann and De Wolff, 1963) *for its purity as we always use the γ-UO*_*3*_ *as the initial compound in the HT/HP synthesis.*

### 2.1 Crystal growth

All the titled compounds were synthesized in the form of small single crystals using the high-temperature/high-pressure solid-state method. All the chemicals were obtained from commercial sources as analytically pure and used without further purification.

**K**_**2**_ **[(UO**_**2**_ **) (Te**_**2**_ **O**_**7**_ **)]**. Uranium trioxide UO_3_ (20.0 mg, 0.0699 mmol), KNO_3_ (21.2 mg, 0.208 mmol), TeO_2_ (22.3 mg, 0.140 mmol), and H_6_TeO_6_ (64.2 mg, 0.279 mmol) in a molar ratio of UO_3_: KNO_3_: TeO_2_: H_6_TeO_6_ = 1 : 3: 2 : 4 were mixed together and finely ground. Then, the mixture was filled into a platinum capsule (outer diameter: 4 mm, wall thickness: 0.2 mm, length:7 mm). The capsule was sealed on both sides with an impulse micro welding device (Lampert PUK U4) and placed into the center of a 1/2-inch piston cylinder talc-pyrex assembly. After this, the capsule was inserted into a 6 mm diameter MgO spacer and positioned in the center of a tapered graphite furnace. The final run pressure of 3.5 GPa was applied within 30 min, then the temperature program was started. With a heating rate of 100 K min^-1^ the temperature was increased to the maximum temperature of 1173 K. After 1 h of annealing, the temperature was decreased to 570 K over a time period of 106 h (cooling rate 0.11 K min^-1^). At 570 K, the experiment was automatically quenched to room temperature. After decompression for 20 min, the capsule was extracted out of the high-pressure assembly and broken. The product of yellow crystals were picked up for further analysis. The yield was impossible to be determined due to the similarity of broken glass pieces and obtained crystals.

**Mg[(UO**_**2**_ **) (TeO**_**3**_ **)**_**2**_ **].** For the synthesis of Mg [(UO_2_) (TeO_3_)_2_], UO_3_ (20.0 mg, 0.0699 mmol), Mg(NO_3_)_2_ (20.7 mg, 0.140 mmol), TeO_2_ (33.5 mg, 0.211 mmol), and H_6_TeO_6_ (16.1 mg, 0.070 mmol) were weighed with a molar ratio of 1:3:1:2 and subsequently thoroughly ground before being filled into platinum capsule. The operations of sealing the platinum capsule and opening it after the reactions are same as mentioned above for the synthesis of K_2_ [(UO_2_) (Te_2_O_7_)]. The pressure of 3.5 GPa was used within 30 min, then the temperature program was started. It was heated up to 1373 K with a heating rate of 100 K min^-1^. After a holding time of 4 h at 1373 K, the temperature was decreased to 1173 K in 1h, and then cooled to 623 K over a time period of 90 h (cooling rate 0.10 K min^-1^). At 623 K, the experiment was automatically quenched to room temperature. After decompression for 20 min, the capsule was extracted out of the high-pressure assembly and broken. The product containing small yellow crystals were picked up for further analysis.

**Sr[(UO**_**2**_ **) (TeO**_**3**_ **)**_**2**_ **] and Sr[(UO**_**2**_ **) (TeO**_**5**_ **)].** Both phases co-precipitated using a finely ground mixture of UO_3_ (30.0 mg, 0.105 mmol), Sr(CO_3_) (15.5 mg, 0.105 mmol), TeO_2_ (16.7 mg, 0.105 mmol), and H_6_TeO_6_ (24.1 mg, 0.0.105 mmol) with a molar ratio of 1:1:1:1. The pressure of 3.5 GPa was applied within 30 min, then the temperature program was started. It was heated up to 1273 K with a heating rate of 100 K min^-1^. After a holding time of 4 h at 1273 K, the temperature was cooled down to 1073 K in 1h, and then decreased to 573 K over a time period of 50 h (cooling rate 0.17 K min^-1^). At 573 K, the experiment was automatically quenched to room temperature. After decompression for 20 min, the capsule was extracted out of the high-pressure assembly and broken. The product containing small yellow crystals together with colorless remains of the educts, mainly consisting of SrCO_3_. The small yellow crystals were picked up for further analysis.

### 2.2 Crystallographic studies

Single crystal X-ray diffraction data for all four compounds were collected on an Agilent Technologies SuperNova diffractometer with Mo-K*α* radiation (*λ* = 0.71073 Å) at room temperature. All data sets were corrected for Lorentz and polarization factors as well as for absorption by the multi-scan method (Sheldrick, 1998). The structures of all four compounds were solved by the direct method and refined by a full-matrix least-squares fitting on *F* ^2^ by SHELX (Almond and Albrecht-Schmitt, 2002; Xiao et al., 2016a). Their structures were checked for possible missing symmetry elements using PLATON with the ADDSYM algorithm, and no higher symmetry was found (Spek, 2001). Crystallographic data and structural refinements for all compounds are summarized in Table 1. More information of the important bond distances and angles of both compounds are listed in Supplementary Table S1. The crystal structures were deposited to the CCDC with the following numbers CSD2238172, CSD2238173, CSD2238174, CSD2238175 for the K_2_ [(UO_2_) (Te_2_O_7_)], Mg [(UO_2_) (TeO_3_)_2_], Sr [(UO_2_) (TeO_3_)_2_] and Sr [(UO_2_) (TeO_5_)], respectively.

**TABLE 1:** Crystallographic data for K_2_ [(UO_2_) (Te_2_O_7_)], Mg [(UO_2_) (TeO_3_)_2_], Sr [(UO_2_) (TeO_3_)_2_] and Sr [(UO_2_) (TeO_5_)].

Compound	K_2_ [(UO_2_) (Te_2_O_7_)]	Mg [(UO_2_) (TeO_3_)_2_]	Sr [(UO_2_) (TeO_3_)_2_]	Sr [(UO_2_) (TeO_5_)]
FW	1,428.62	645.53	708.84	565.24
Space group	*C2/c*	*Cmca*	*Pbam*	*Pbam*
*a* Å)	10.76408)	7.58644)	11.5299 (12)	12.53772)
*b* Å)	10.1671 (12)	11.51725)	7.89319)	12.45992)
*c* Å)	7.1707 (10)	7.51433)	4.03614)	7.57807 (16)
*β* (deg)	93.101 (10)	90	90	90
*V* (Å^3^)	783.60 (15)	656.555)	367.316)	1,183.834)
Z	4	4	2	8
*λ*(Å)	0.71073	0.71073	0.71073	0.71073
F (000)	1,224	1,088	1,476	1955
*D*_*c*_ (g cm^-3^)	6.064	6.531	6.409	6.506
GOF on *F* ^2^	1.081	1.035	1.085	1.093
*R*_1_	0.0201	0.0267	0.0393	0.0214
*w*R2	0.0561	0.0695	0.0900	0.0710
$R1=\sum \left|\left|F_{o}\right|-\left|F_{c}\right|\right|/\sum \left|F_{o}\right|,wR2={\left\{\sum w{\left[{\left(F_{o}\right)}^{2}-{\left(F_{c}\right)}^{2}\right]}^{2}/\sum w{\left[{\left(F_{o}\right)}^{2}\right]}^{2}\right\}}^{1/2}$				

### 2.3 Bond-valence analysis

As a semi-empirical method for the approximate determination of valence states, BVS of all atoms in both phases were calculated. The bond-valence parameters for U(VI)-O, K(I)-O, Mg(II)-O, Sr(II)-O, Te(IV)-O, Te(V)-O and Te(VI)-O were used according to Burns (Burns et al., 1997), Brese and O’Keeffe (Brown and Altermatt, 1985; Brese and O’Keeffe, 1991).

## 3 Results and discussion

### 3.1 Crystal growth

The investigation of the *A*-U-Te-O (*A* = alkali and alkaline Earth metal) system under extreme HT/HP conditions (3.5GPa, 1173–1373 K) yielded four novel compounds: K_2_ [(UO_2_) (Te_2_O_7_)], Mg [(UO_2_) (TeO_3_)_2_], Sr [(UO_2_) (TeO_3_)_2_] and Sr [(UO_2_) (TeO_5_)]. K_2_ [(UO_2_) (Te_2_O_7_)] was obtained through the usage of UO_3_: KNO_3_: TeO_2_: H_6_TeO_6_ in a ratio of 1 : 3: 2 : 4. Although analytical-grade Te^ⅠV^O_2_ and H_6_Te^VⅠ^O_6_ were used as an initial reagent, interestingly, tellurium occurs as Te^V^ with a 5+ oxidation state in K_2_ [(UO_2_) (Te_2_O_7_)]. We concluded that Te^ⅠV^O_2_ and H_6_Te^VⅠ^O_6_ undergo an oxidation-reduction chemical reaction opposite to the disproportionation of Te^V^ under the extreme conditions (3.5GPa, 1173 K). Attempts were made to synthesize K_2_ [(UO_2_) (Te_2_O_7_)] through high temperature solid state reaction at ambient pressure, however this was unsuccessful. This suggests the high pressure is an essential factor for this redox reaction. For the synthesis of Mg [(UO_2_) (Te^ⅠV^O_3_)_2_]: UO_3_, Mg(NO_3_)_2_, TeO_2_ and H_6_TeO_6_ were taken in molar ratios of 1 : 2: 3:1. Whereas both phases of Sr [(UO_2_) (Te^ⅠV^O_3_)_2_] and Sr [(UO_2_) (Te^VⅠ^O_5_)] co-precipitated using a finely ground mixture of UO_3_, Sr(CO_3_), TeO_2_ and H_6_TeO_6_ with a molar ratio of 1:1:1:1. We presumed that the ratios of TeO_2_ and H_6_TeO_6_ were used in the original reagents has also played a key role for the final oxidation states of tellurium in the compounds. The obtained materials have been found in the form of relatively small single crystals (up to 1 mm size). We presume that they grow up within so called self-flux which is usual for the materials crystallization from multicomponent high-temperature solid-state systems (Balraj and Vidyasagar, 1999; Kim et al., 2007a; Kim et al., 2010).

### 3.2 Crystal structures

#### 3.2.1 Structure of K_2_[(UO_2_) (Te_2_O_7_)]

**FIGURE 1:**
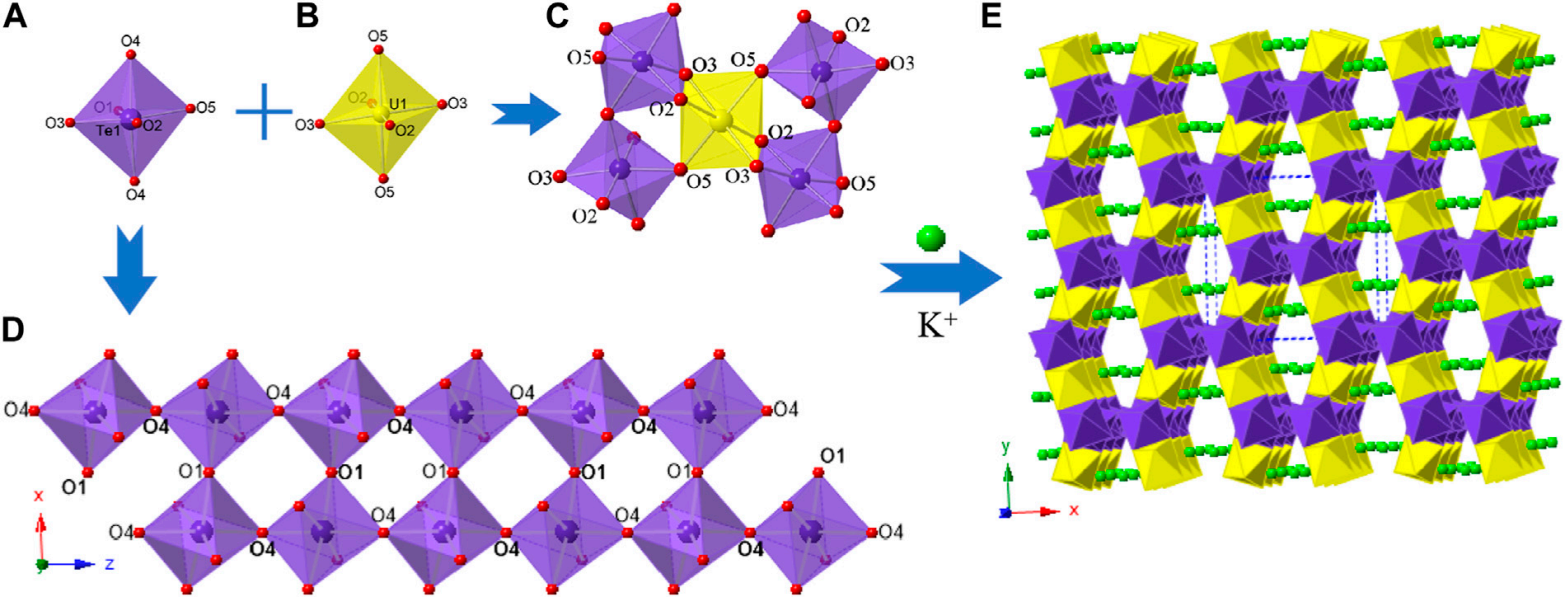
K_2_ [(UO_2_) (Te_2_O_7_)]: **(A)** TeO_6_ polyhedron; **(B)** UO_6_ polyhedron, **(C)** coordination environment for UO_6_; **(D)** a Te_-_O double chain along the *c*-axis; and **(E)** view of the 3D structure along the *c*-axis. K atoms, TeO_6_ polyhedra, UO_6_ polyhedra, and O atoms are shown in green, purple, yellow and red, respectively.

**FIGURE 2:**
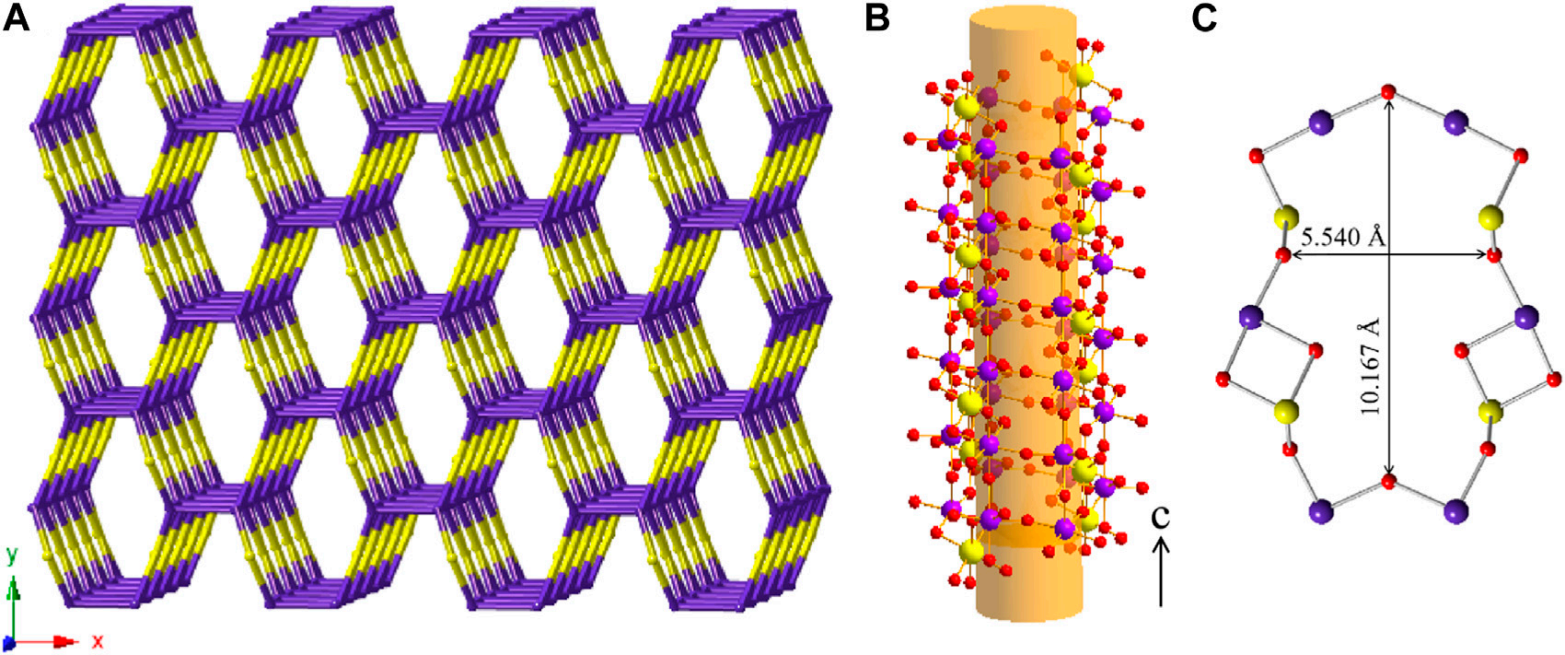
**(A)** View of the cation framework topology representation of K_2_ [(UO_2_) (Te_2_O_7_)] along the *c*-axis; **(B)** Side view of one 10-MRs tube along the [001] direction with ball-and-stick representation; **(C)** the pore size of a 10-MRs of the [(UO_2_)Te_2_O_7_] tube. K, Te, U and O atoms are shown in green, purple, yellow and red, respectively.

K_2_ [(UO_2_) (Te_2_O_7_)] crystallizes in the monoclinic space group *C*2/c. The structure of K_2_ [(UO_2_) (Te_2_O_7_)] can be described as a 3D open framework composed of uranium and tellurium polyhedra (Figure 1). Its framework is featured with 1D [Te_2_O_7_]^4-^ double-chains, formed by a vertex (O4 and O1) sharing linkage along the *c*-axis (Figure 1D). These [Te_2_O_7_]^4-^ double-chains are further connected by UO_6_ polyhedra, forming the 3D [(UO_2_) (Te_2_O_7_)]^2-^ anionic framework (Figure 1C; Figure 1E). Relatively large 1D tunnels with 10-MRs (10.167 Å ×5.540 Å) can be observed in the structure along the *c*-axis (Figure 2). K^+^ cations fill the channels to balance the charge of the framework.

There is one crystallographically unique tellurium site Te (1), one uranium site U (1), one potassium site K (1) and five oxygen sites O (1)-O (5) in the structures of K_2_ [(UO_2_) (Te_2_O_7_)], respectively. Corner-shared TeO_6_ octahedra form a one-dimensional double-chain along the *c*-axis, and these chains are further connected by UO_6_ polyhedra along the *b*-axis, resulting in the 3D network (Figure 1E). The UO_6_ pseudooctahedral coordination alternate between the Te-O double chains and are corner-shared (O5) to two TeO_6_ octahedra and edge-shared (O2-O3) to two TeO_6_ octahedra. The Te-O bond distances in TeO_6_ octahedra range between 1.888(4) and 1.979(4) Å, whereas the U-O bond distances are in the range of [1.992(4)-2.031(4) Å]. Potassium cations are 10-oxygen coordinated and K-O bond distances range from 2.676(5) to 3.304(5) Å. All the bond angles and bond lengths in K_2_ [(UO_2_) (Te_2_O_7_)] are as shown in Supplementary Table S1. These bond lengths are comparable with previously reported works (Almond and Albrecht-Schmitt, 2002; Chi et al., 2006; Kim et al., 2007b; Kim et al., 2014; Xiao et al., 2016a). From BVS calculations, the valences for U cations are suggested to be 6+ with values for the U1 at ca. 6.11. The valences for K cations are 1+ with BVS values for K1 at ca. 1.25.

It is noteworthy to compare the structure of K_2_ [(UO_2_) (Te_2_O_7_)] with the series of *A* [CuTe_2_O_7_] (A = Sr, Ba, or Pb) and Ba [*M*Te_2_O_7_] (*M* = Mg or Zn) (Yeon et al., 2011; Yeon et al., 2012), due to them having similar chemical composition with isovalent cations substituting UO_2_^2+^ groups through divalent cations. Although K_2_ [(UO_2_) (Te_2_O_7_)], *A* [CuTe_2_O_7_] (A = Sr, Ba, or Pb) and Ba [*M*Te_2_O_7_] (*M* = Mg or Zn) have same stoichiometries, the structure of K_2_ [(UO_2_) (Te_2_O_7_)] is different from that series. In K_2_ [(UO_2_) (Te_2_O_7_)], Te atoms occur as Te^V^ as TeO_6_ polyhedra, whereas in *A* [CuTe_2_O_7_] (A = Sr, Ba, or Pb) and Ba [*M*Te_2_O_7_] (*M* = Mg or Zn) they are mixed valent tellurium, Te^4+^ and Te^6+^. SrCuTe_2_O_7_ and PbCuTe_2_O_7_ are isostructural, and their two-dimensional crystal structure consists of 2D layers based upon corner-sharing CuO_5_ square pyramids, TeO_6_ octahedra, and TeO_4_ dispheniods. Ba*M*Te_2_O_7_ (*M* = Mg^2+^ and Zn^2+^) are iso-structural with BaCuTe_2_O_7_, and exhibit a crystal structure composed of layers of corner-shared *M*O_5_ (*M* = Mg^2+^ or Zn^2+^) square pyramids, TeO_6_ octahedra, and TeO_4_ polyhedra. The [MTe_2_O_7_]^2−^ anionic layers (*M* = Mg^2+^ and Zn^2+^) stack along the *b*-axis, and are separated by Ba^2+^ cations. Comparing these phases we can presume that high-pressure conditions applied in our study not only condensed the final structures (from 2D towards 3D), but also influenced the redox stability of Te with stabilization of the rare Te^V^ cation.

**FIGURE 3:**
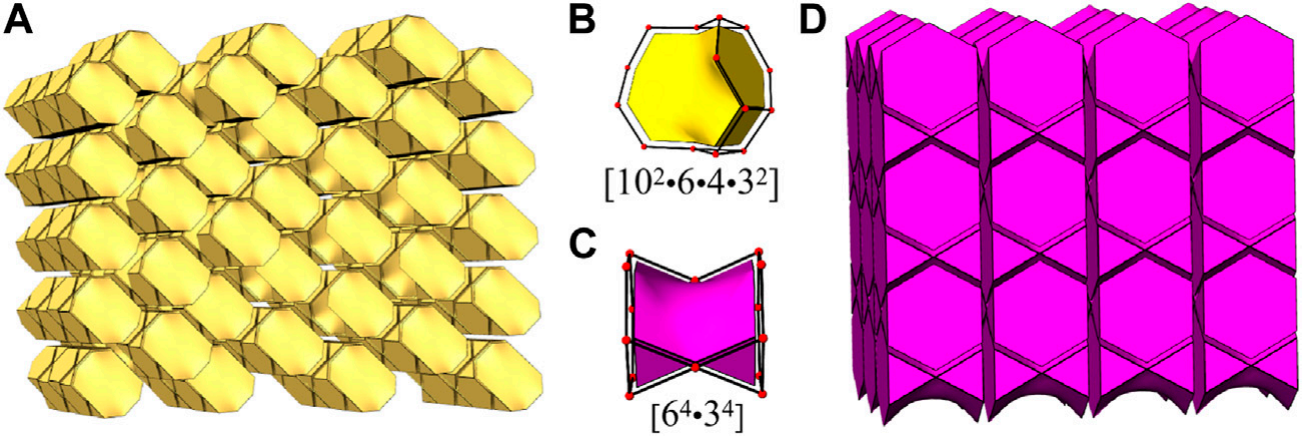
View of the channel systems in K_2_ [(UO_2_) (Te_2_O_7_)] **(A)** and Sr [(UO_2_) (TeO_5_)] **(D)** using natural tiling, new *CBU*s [10^2^?6?4?3^2^] **(B)** and [6^4^?3^4^] **(C)**.

In order to reveal the complex topological network of K_2_ [(UO_2_) (Te_2_O_7_)], we simplify the anionic uranyl tellurium framework [(UO_2_)Te_2_O_7_]^2-^, by removing the oxygen atoms, whilst the UO_6_ and TeO_6_ polyhedra were viewed as single nodes. As shown in Figure 2A, the simplified anionic net of K_2_ [(UO_2_) (Te_2_O_7_)] can be described as a 2-nodal net topological type with a point symbol of {3^2^.4^2^.5^2^.6^3^.7}_2_{3^2^.6^2^.7^2^} (Blatov, 2006; Blatov et al., 2010; Alexandrov et al., 2011), which is a 4, 5-*c* net with stoichiometry (4-c) (5-c)_2._ Natural tiling is an efficient approach to represent a network proposed by Blatov *et al.* (Blatov et al., 2007), which can be used for illustrating the channel system and cavities by tracing the colors of the tiles clearly as shown in Figure 3A. The framework of K_2_ [(UO_2_) (Te_2_O_7_)] is built from a novel composite building unit (*CBU*) [10^2^:6:4:3^2^] (Figure 1B), with the 10-MR tunnels along the *c*-axis. Each [10^2^:6:4:3^2^] *CBU* connects to four other neighboring ones, *via* their 10, 6, four or 3-MRs defining the 3D tiling network. Compared with the previously reported uranyl borates, phosphates and borophosphates system, the tilting network of this uranyl tellurium system is simpler with only one unique *CBU*. (Burns et al., 1997; Hao et al., 2016; Hao et al., 2017a; Hao et al., 2017b; Hao et al., 2018; Hao et al., 2020a; Hao et al., 2022), (Hao et al., 2013; Hao et al., 2014; Hao et al., 2020b), (Hao et al., 2020c; Hao et al., 2020d; Li et al., 2022) Similar situation is for the structure of Sr [(UO_2_) (TeO_5_)] (see below).

#### 3.2.2 Structure of Mg[(UO_2_) (TeO_3_)_2_]

**FIGURE 4:**
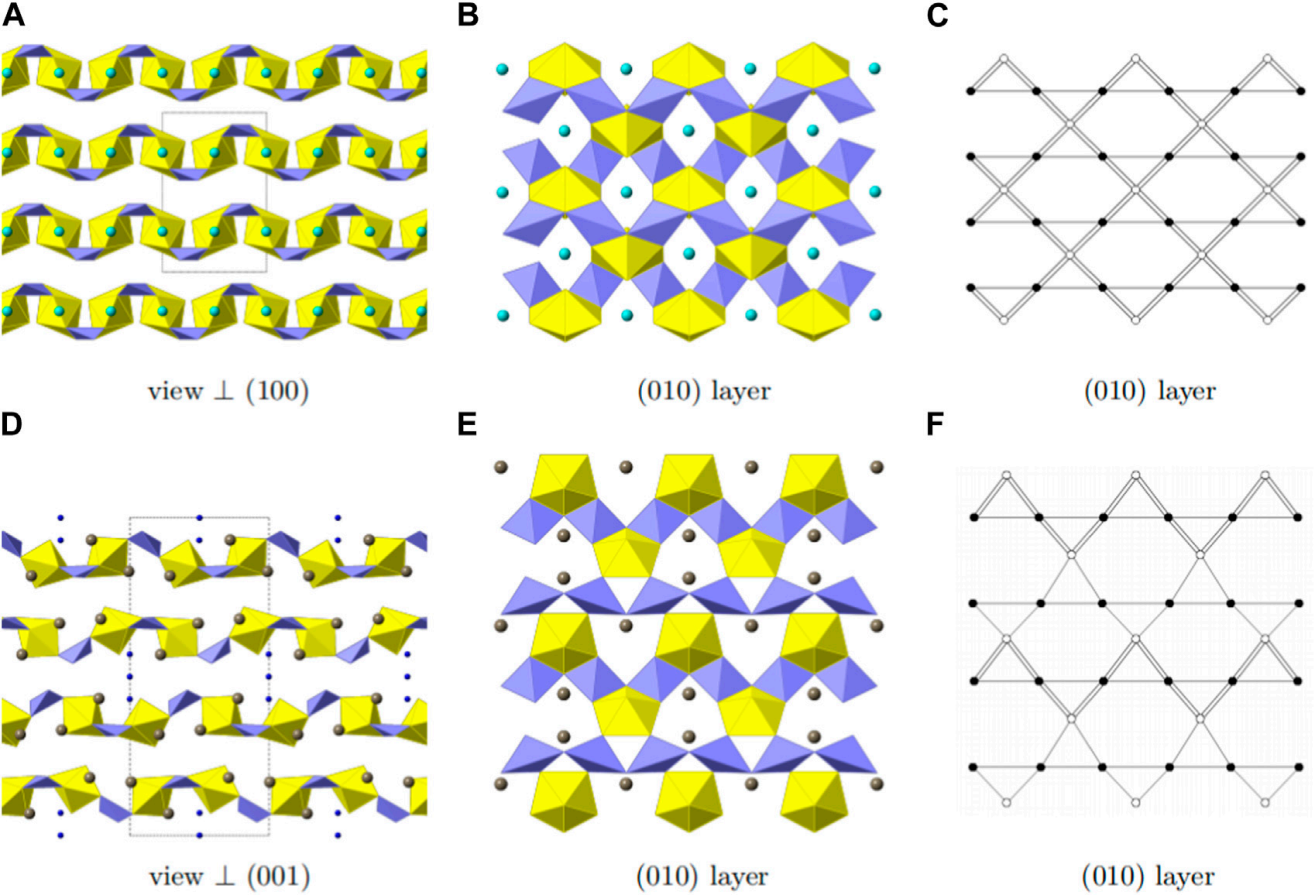
Structural and topologic depiction of Mg [(UO_2_) (TeO_3_)_2_] **(A–C)** and in comparison Tl_3_ ((UO_2_)_2_ (Te_2_O_5_(OH)) (Te_2_O_6_))?2(H_2_O) **(D–E)**. In **(A)** and **(D)** a side view of the structure of the former and the latter can be seen. The unit-cell outline is shown in dashed-lines. In Mg [(UO_2_) (TeO_3_)_2_], an *ABAB* ordering principle is visible whereas an *ABCDABCD* is found in Tl_3_ ((UO_2_)_2_ (Te_2_O_5_(OH)) (Te_2_O_6_))·2(H_2_O). In **(B)** and **(E)** a single (010) layer for both structures is shown and the according topology is depicted in **(C)** and **(F)**, respectively. UO_7_ polyhedra are depicted in yellow, TeO_4_ polyhedra in light purple, Mg in cyan, Tl in grey and water molecules in deep blue.

Mg [(UO_2_) (TeO_3_)_2_] crystallizes in the orthorhombic space group *Cmca*. Mg, U and Te occupy one crystallographically independent position each and for oxygen three crystallographically independent positions are occupied. As shown in Figure 4, Uranium is eight fold-coordinated by oxygen in bipyramidal fashion. The two oxygen positions located at the pyramid tops have a U-O bond length of 1.805(6) Å. Together with a bond angle of 180.0° between O-U-O along the central axis, these are typical values for a uranyl group. The equatorial oxygen atoms adopt bond-distances from 2.361(7) to 2.527(5) Å.

Tellurium is coordinated by four oxygen atoms resulting in disphenoidal symmetry. The electron lone pair points towards the top center of the disphenoid. The bond distances range from 1.881(4) to 2.042(3) Å. This coordination is found to be common within Te^IV^ structures (Balraj and Vidyasagar, 1999; Xiao et al., 2016b).

In Mg [(UO_2_) (TeO_3_)_2_], TeO_4_ disphenoids share common corners with each other resulting in infinite onedimensional chains of [(TeO_3_)_2_]^4−^ propagating along the a-axis. These chains link the uranyl bipyramids by edge sharing along two edges of the disphenoids. This results in two-dimensional sheets of [(UO_2_) (Te_2_O_6_)]^2−^ (Figure 4B). Within these sheets, four UO_8_ hexagonal bipyramids and four TeO_4_ disphenoids form a ring structure. This is well visible in the topologic description shown in Figure 4C.

Mg^2+^ are located within the rings of UO_8_ and TeO_4_ mentioned above. It is coordinated by six oxygen atoms in distances ranging from 2.051(5) to 2.284(6) Å leading to a rectangular bipyramid. Mg^2+^ acts as a counter charge for the [(UO_2_) (Te_2_O_6_)]^2−^ layers-resulting in a neutrally charged phase. Bond valence sum calculations for all positions (Mg∼ 2.03, U∼5.98, Te∼4.06) yield values in accordance to the assumed oxidation states.

The layers found in Mg [(UO_2_) (TeO_3_)_2_] most resemble the layers found in Tl_3_ ((UO_2_)_2_ (Te_2_O_5_(OH)) (Te_2_O_6_))·2(H_2_O) (Almond and Albrecht-Schmitt, 2002). The layers found in the latter are depicted in Figure 4E. Both structures possess one-dimensional chains of corner-sharing disphenoidal coordinated Te. Due to the presence of Mg^2+^ and Tl^1+^, both structures are electro-neutral despite the charged layers. The essential difference between the two structures, arises from the presence of OH^−^ groups in Tl_3_ ((UO_2_)_2_ (Te_2_O_5_(OH)) (Te_2_O_6_))·2(H_2_O) in comparison to their absence in Mg [(UO_2_) (TeO_3_)_2_]. In the former, two different chains of corner-sharing disphenoidal coordinated Te are present [(Te_2_O_5_(OH)]^3-^ and [(TeO_3_)_2_]^4-^. In Mg [(UO_2_) (TeO_3_)_2_] only chains of [(TeO_3_)_2_]^4-^ are present. This leads to constant edge-sharing connections between tellurite disphenoids and uranyl bipyramids in Mg [(UO_2_) (TeO_3_)_2_], whereas edge-sharing connections are only found along the [(TeO_3_)_2_]^4-^-chains in Tl_3_ ((UO_2_)_2_ (Te_2_O_5_(OH)) (Te_2_O_6_))·2(H_2_O) and corner-sharing positions along the [(Te_2_O_5_(OH)]^3−^ chains. The difference of the resulting layers can also clearly be seen, whilst comparing the topology graphs for both structures. (Figures 4C,F).

Due to the different connections, the uranyl bipyramids in Tl_3_ ((UO_2_)_2_ (Te_2_O_5_(OH)) (Te_2_O_6_))·2(H_2_O) are only pentagonal whereas they are hexagonal in Mg [(UO_2_) (TeO_3_)_2_]. This is the first report of hexagonal bipyramids in two-dimensional phases containing U and Te. Otherwise, these are only found in three-dimensional phases, such as within UTe_3_O_9_ (Galy and Meunier, 1971) or Na [(UO_2_)Te_6_O_13_(OH)] (Almond and Albrecht-Schmitt, 2002; Xiao et al., 2016a). It is also worth mentioning, that both phases have different packing orders of their layers. In Mg [(UO_2_) (TeO_3_)_2_] only two layers are stacked until the original layer is repeated (*ABAB* stacking), whereas four layers are necessary in Tl_3_ ((UO_2_)_2_ (Te_2_O_5_(OH)) (Te_2_O_6_))·2(H_2_O) (*ABCDABCD* stacking) (Figures 4A,D).

#### 3.2.3 Structure of Sr[(UO_2_) (TeO_3_)_2_]

**FIGURE 5:**
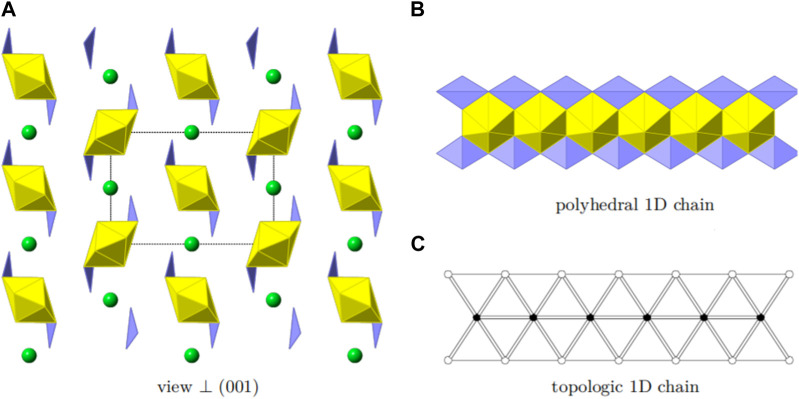
Structural and topologic depiction of Sr [(UO_2_) (TeO_3_)_2_]. In **(A)** a side view of the structure can be seen. The unit-cell outline is shown in dashed-lines. In **(B)**, chains of [(UO_2_) (TeO_3_)_2_]^2−^ propagating into the [001] direction are shown. The according topology is depicted in **(C)**.

Sr [(UO_2_) (TeO_3_)_2_] crystallizes in the orthorhombic space group *Pbam*. U, Sr and Te each are present on one independent position. As U and Sr lie on special positions (0.5, 0.5, 0.5 and 0.5, 0.0, 0.5, respectively) and Te lies on a general position in respect to *x* and *y* (0.2894, 0.2644, 0.0), the multiplicity results in a U:Te molar ratio of 1:2. Oxygen is present on four independent positions, each of them are general positions. The structure of Sr [(UO_2_) (TeO_3_)_2_] is depicted in Figure 5A.

Uranium is coordinated by two short-bonded actinyl oxygen with <U-O_yl_> = 1.825 (13) Å and six equatorial oxygen (<U-O_eq_> = 2.349(7) Å) forming hexagonal bipyramids and thus hexavalent U is present. BVS calculations are well in agreement with 6.21 *v. u.* Te is coordinated by four oxygen atoms forming a disphenoidal coordination polyhedron. This coordination is typical for tetravalent Te and BVS calculations yield 4.01 *v. u.*, supporting the assignment for tetravalent Te. The bonding distances range from 1.849 (13) to 2.102(4) Å. Eight oxygen positions surround Sr with bond distances of 2.525(7) to 3.029 (14) Å. Sr is predominantly stable as divalent Sr and a BVS of 2.09 *v. u*. is well in agreement with this.

The structure is based on one-dimensional chains of [(UO_2_) (TeO_3_)_2_]^2−^ propagating into the [001] direction. These chains are formed by edge-sharing uranyl bipyramids which are additionally fused together by two TeO_4_ disphenoids, edge-sharing along two edges. Such a chain is depicted in Figure 5B. The according topology is shown in Figure 5C. The charge of the chains are compensated by the presence of the Sr^2+^ cations. The apparent additional void space is filled by the electron-lone pairs from the tetravalent Te. These are directed along the [010].

It is worth comparing the structures of chemical analogues Mg [(UO_2_) (TeO_3_)_2_] and Sr [(UO_2_) (TeO_3_)_2_]. In Mg [(UO_2_) (TeO_3_)_2_], TeO_4_ disphenoids share common corners with each other resulting in infinite 1D chains of [(TeO_3_)_2_]^4-^ propagating along the *a*-axis. These chains link the uranyl bipyramids by edge sharing along two edges of the disphenoids, resulting in the 2D layered structure of [(UO_2_) (Te_2_O_6_)]^2-^. Mg^2+^ counter cations are located between the inter-layers to balance the anionic layered charge. The structure of Sr [(UO_2_) (TeO_3_)_2_] features a 1D chain structure [(UO_2_) (TeO_3_)_2_]^2-^, propagating into the *c*-axis. These chains are formed by edge-sharing uranyl bipyramids which are additionally fused together by two TeO_4_ disphenoids, which also share two edges. We can see that with the counter cations radii increasing from Mg^2+^ to Sr^2+^ this results in the structure of materials changing from a 2D to a 1D structural type. In this case we can speak of a morphotropic transition within A^II^ [(UO_2_) (TeO_3_)_2_] (A^II^- alkali-earth elements).

#### 3.2.4 Structure of Sr[(UO_2_) (TeO_5_)]

Sr [(UO_2_) (TeO_5_)] crystallizes in the orthorhombic space group P*bam*. The crystallographic data is given in Table 1. It forms a 3D framework structure made up of UO_7_ and TeO_6_ polyhedra with Sr^2+^ cations filling the voids to achieve charge neutrality.

Two uranium positions are present within the structure and both have a typical bipyramidal pentagonal oxygen coordination of UO_7_. The bond lengths of the uranyl oxygen positions are < U1-O_yl_> = 1.81598) Å and <U2-O_yl_> = 1.822(7) Å. This can be explained by the dense packing of the framework leading to a stronger coordination of the equatorial plane with average bond lengths of <U1-O_eq_> = 2.353(7) Å and <U2-O_eq_> = 2.357(1) Å, respectively. The closer coordination of the equatorial oxygen positions is charge compensated by the uranyl bonds. Both, U1O_7_ and U2O_7_, are not interconnected to each other, are however interlinked by octahedral TeO_6_ units. The resulting framework is described in more detail below.

Te only adopts a single position and is coordinated by six oxygen atoms to form distorted octahedral polyhedra (Supplementary Figure S1). Hereby, two positions are slightly elongated (O1 and O4) with Te-O1 = 1.975(2) Å and Te-O4 = 1.980(2) Å. The other four positions range from 1.8805) Å to 1.9802) Å. Two TeO_6_ polyhedra are interconnected by corner sharing of the O1 and O4 positions, leading to one-dimensional chains propagating in the [001] direction.

**FIGURE 6:**
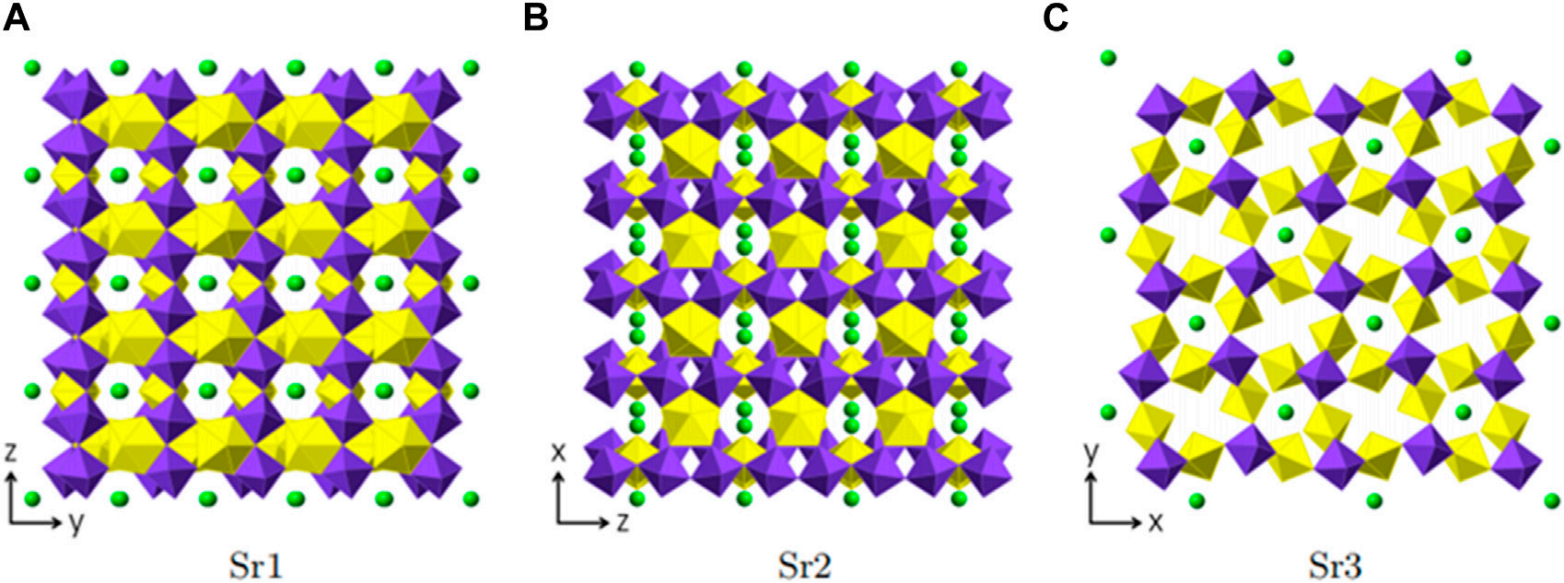
Sr positions within Sr [(UO_2_) (TeO_5_)] depicted in green. Each figure shows the complete uranyl tellurate framework with only the according Sr^2+^ positions depicted. **(A)** Srl -view ⊥[100], **(B)** Sr2-view⊥[010] and **(C)** Sr3-view⊥[001].

**FIGURE 7:**
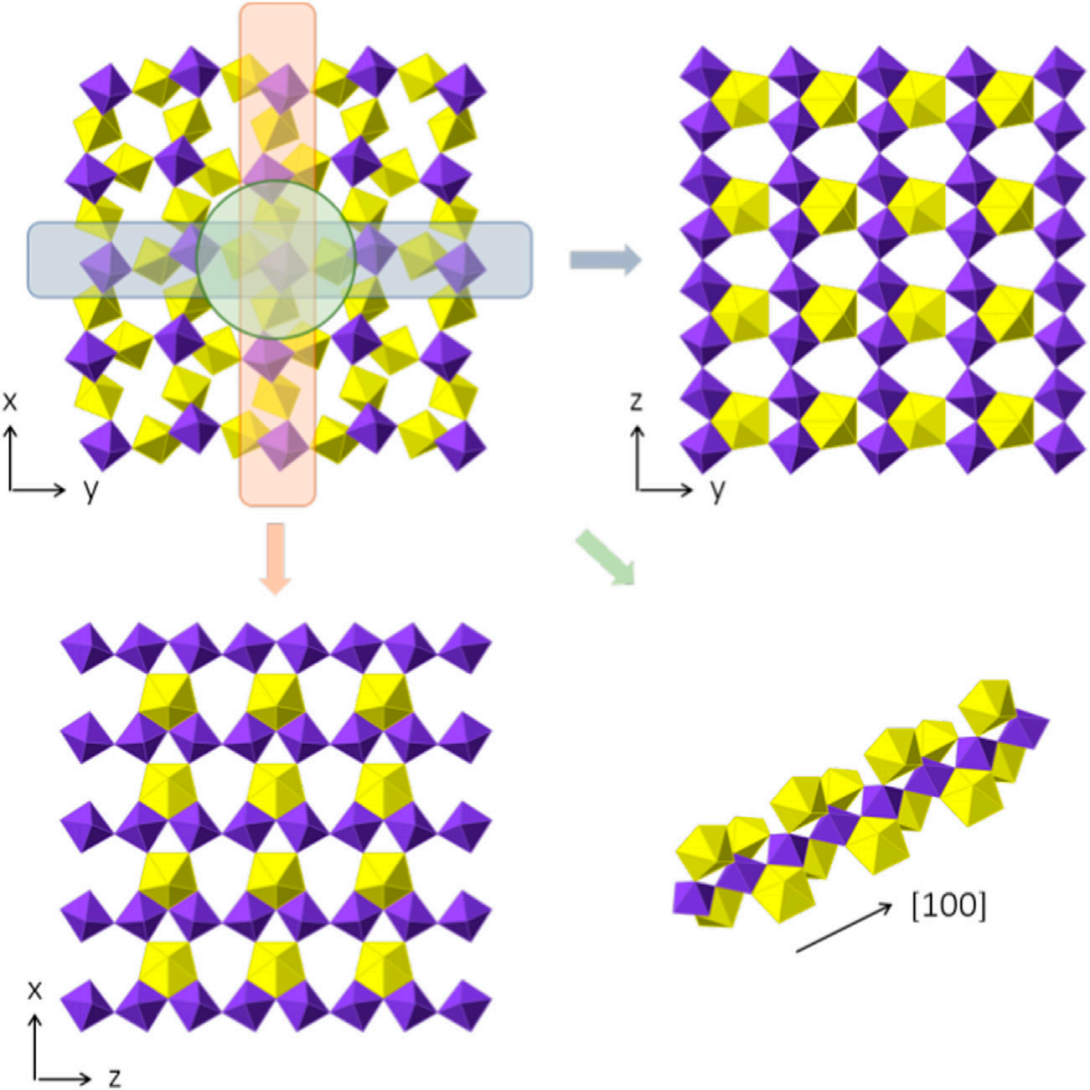
Framework composition of Sr [(UO_2_) (TeO_5_)]. In the top-left, the complete structure, omitting the Sr positions for clarity, which are positioned within the empty voids, is shown, perpendicular to [001]. Two-dimensional slices of the (100) and (010) plane are shown on the top right and the lower left. The layers are basically identical, despite the orientation. In the lower right, the infinite chain of [TeO_5_]^4−^ polyhedra is shown with the corner and edge sharing with UO_7_ pentagonal bipyramids.

Three crystallographically independent Sr positions are present in Sr [(UO_2_) (TeO_5_)]. All three positions are shown in Figures 6A–C. Sr1 is located within [100] channels and coordinated tenfold by oxygen with distances ranging from 2.460(5) Å to 3.175(7) Å. Sr2 and Sr3 are eight-fold coordinated with distances ranging from 2.429(5) Å to 2.985(9) Å and 2.454(5) Å to 2.935(5) Å, respectively. Sr2 is located within [010] channels and Sr3 within [001] channels. To describe the framework structure of Sr [(UO_2_) (TeO_5_)], it is best to divide the structure into simpler one and two-dimensional units. This is shown in Figure 7. As already stated above, the structure is made up of infinite [TeO_5_]^4−^ chains. Two opposite edges of the TeO_6_ octahedron are involved in edge-sharing with U2O_7_ and U1O_7_ bipyramids and the two remaining oxygen positions each corner-share with a U1O_7_ and a U2O_7_ (Supplementary Figure S1). The same framework topology can be found in Na_2_ [(UO_2_) (TeO_5_)] (Almond and Albrecht-Schmitt, 2002; Xiao et al., 2016a).

**FIGURE 8:**
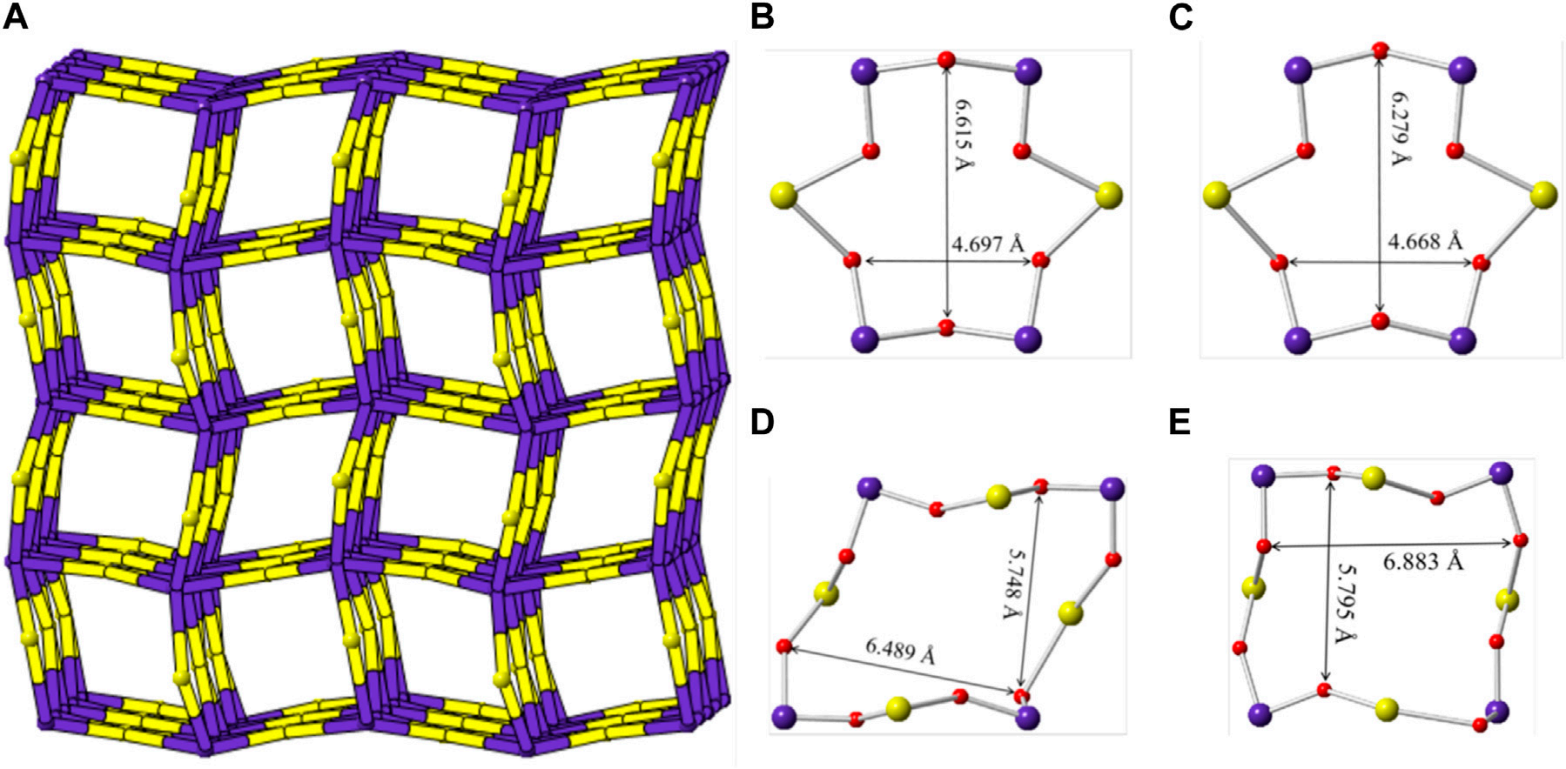
**(A)** View of the cation framework topology representation of Sr [(UO_2_) (TeO_5_)] along the *c*-axis; **(B**–**E)** the 6-MRs and 8-MRs pore size of the [(UO_2_) (TeO_5_)] tube along the *a*-, *b*- and *c*-axis. K, Te, U and O atoms are shown ain green, purple, yellow and red, respectively.

Sr [(UO_2_) (TeO_5_)] has a multi-intersecting channel system as shown in Figure 8. Two 6-MRs channels exist along the *a*- and *b*-axis with diameters of ca. 6.6 Å × 4.7 Å and ca. 6.3 Å × 4.7 Å (distances are based on two opposite O atoms), respectively. (Figures 8B,C). Two 8-MRs channels is along the *c*-axis with diameters of ca. 6.5 Å × 5.7 Å and ca. 6.9 Å × 5.8 Å (Figures 8D,E). For the purpose of revealing the complex topological network of Sr [(UO_2_) (TeO_5_)], we also simplified the anionic uranyl tellurium framework [(UO_2_) (TeO_5_)]^2-^, by removal of the oxygen anions, whilst the UO_7_ and TeO_6_ polyhedra were viewed as single nodes. As shown in Figure 8A, the simplified anionic net of Sr [(UO_2_) (TeO_5_)] can be described as a 2-nodal net topological type with a point symbol of {3^2^.6^2^.7^2^}{3^4^.4^2^.6^4^.7^5^}; which is a 4, 6-*c* net with stoichiometry of (4-c) (6-c) (Brese and O’Keeffe, 1991; Blatov et al., 2010; Alexandrov et al., 2011). Natural tiling illustration of Sr [(UO_2_) (TeO_5_)] is shown in Figure 3D. The framework of Sr [(UO_2_) (TeO_5_)] is built from a novel *CBU* of [6^4^⋅3^4^] (Figure 3C), with the 6-MR intersecting tunnels along the corresponding axis. Each [6^4^⋅3^4^] *CBU* connects to eight other neighboring ones, *via* their six or 3-MRs window defining the 3D tiling network.

## 4 Conclusion

We have shown that by using extreme synthetic conditions (high-temperature high-pressure) novel single crystalline phases in the U-Te-O system can be obtained. Four new uranyl tellurium complex oxides obtained in this study, K_2_ [(UO_2_) (Te_2_O_7_)], Mg [(UO_2_) (TeO_3_)_2_], Sr [(UO_2_) (TeO_3_)_2_] and Sr [(UO_2_) (TeO_5_)], featured 1D-chain to 3D-framework structures. Surprisingly, tellurium is occurs as Te^V^ in one of the obtained materials, K_2_ [(UO_2_) (Te_2_O_7_)], which is a rare case in tellurium bearing inorganic phases. Under the same pressure (3.5 GPa) three novel alkaline Earth metal uranyl tellurites/tellurate, Mg [(UO_2_) (TeO_3_)_2_], Sr [(UO_2_) (TeO_3_)_2_] and Sr [(UO_2_) (TeO_5_)], were obtained. Mg [(UO_2_) (TeO_3_)_2_] and Sr [(UO_2_) (TeO_3_)_2_] have the same chemical formulas, but they are not iso-structures. This indicates that with the larger Sr^2+^counter cation the dimensionality of the structure upon the ratio of U:Te = 1:2 decreases. It is noteworthy to consider the HT/HP role in generating these unique 1D to 3D structures, particularly in comparison to other actinide and non-actinide tellurium bearing compounds. Especially, HT/HP plays an important role in the stabilization of the less stable Te^V^ which we were not able to reproduce under normal conditions. This study further demonstrates how subtle adjustments to counter cations can reveal dramatic changes to structural type and topology. Further investigation ofthe *A*-U-Te-O system under similar synthetic conditions will lead towards obtaining different unique types of structures. This will help in achieving a deeper understanding of the influence of extreme conditions on the chemically complex tellurium bearing oxo-phases.

## Data Availability

The datasets presented in this study can be found in online repositories. The names of the repository/repositories and accession number(s) can be found below: https://www.ccdc.cam.ac.uk/structures/- CSD2238172, CSD2238173, CSD2238174, CSD2238175.
