# Supplementary material for: Understanding of the structural chemistry in the uranium oxo-tellurium system under HT/HP conditions
Source: Front Chem. 2023 Mar 10;11:1152113. doi: 10.3389/fchem.2023.1152113 (PMC10037309; doi:10.3389/fchem.2023.1152113)

checkCIF/PLATON report

Structure factors have been supplied for datablock(s) KUO22Te2O7

THIS REPORT IS FOR GUIDANCE ONLY. IF USED AS PART OF A REVIEW PROCEDURE FOR PUBLICATION, IT SHOULD NOT REPLACE THE EXPERTISE OF AN EXPERIENCED CRYSTALLOGRAPHIC REFEREE.

No syntax errors found. CIF dictionary Interpreting this report

Datablock: KUO22Te2O7

```
Bond precision:   U- O = 0.0040 A         Wavelength=0.71073

Cell:             a=10.7640(8)     b=10.1671(12)      c=7.1707(10)
                  alpha=90         beta=93.101(10)    gamma=90
Temperature:      293 K

                Calculated                      Reported
Volume          783.60(15)                      783.60(15)
Space group     C 2/c                           C 2/c
Hall group      -C 2yc                          ?
Moiety formula  O18 Te4 U2, 4(K)                ?
Sum formula     K4 O18 Te4 U2                   K2 O9 Te2 U
Mr              1430.86                         715.43
Dx,g cm-3       6.064                           6.064
Z               2                               4
Mu (mm-1)       29.103                          29.103
F000            1224.0                          1224.0
F000'           1184.12
h,k,lmax        13,12,9                         13,12,9
Nref            864                             859
Tmin,Tmax       0.076,0.130                     0.854,1.000
Tmin'           0.044

Correction method= # Reported T Limits: Tmin=0.854 Tmax=1.000
AbsCorr = MULTI-SCAN

Data completeness= 0.994           Theta(max)= 27.050

R(reflections)= 0.0201( 840)                    wR2(reflections)=
                                                0.0563( 859)
S = 1.081                    Npar= 67
```

The following ALERTS were generated. Each ALERT has the format
test-name_ALERT_alert-type_alert-level.
Click on the hyperlinks for more details of the test.

Alert level C

```
ABSTY02_ALERT_1_C  An _exptl_absorpt_correction_type has been given without
            a literature citation. This should be contained in the
            _exptl_absorpt_process_details field.
            Absorption correction given as multi-scan
PLAT199_ALERT_1_C Reported _cell_measurement_temperature ..... (K)        293 Check
PLAT200_ALERT_1_C Reported   _diffrn_ambient_temperature ..... (K)        293 Check
```

Alert level G

```
PLAT003_ALERT_2_G Number of Uiso or Uij Restrained non-H Atoms ...          1 Report
PLAT004_ALERT_5_G Polymeric Structure Found with Maximum Dimension          2 Info
PLAT005_ALERT_5_G No Embedded Refinement Details Found  in the CIF     Please Do !
PLAT045_ALERT_1_G Calculated and Reported Z Differ by a Factor ...      0.500 Check
PLAT083_ALERT_2_G SHELXL Second Parameter in WGHT  Unusually Large      14.55 Why ?
PLAT395_ALERT_2_G Deviating  X-O-Y   Angle From 120 for O1       .      131.8 Degree
PLAT395_ALERT_2_G Deviating  X-O-Y   Angle From 120 for O4       .      133.6 Degree
PLAT710_ALERT_4_G Delete 1-2-3 or 2-3-4 Linear Torsion Angle ... #          7 Do !
              TE1 -U1  -TE1 -O5     36.00  3.00   7_556   1_555   1_555   1_555
PLAT710_ALERT_4_G Delete 1-2-3 or 2-3-4 Linear Torsion Angle ... #         17 Do !
              TE1 -U1  -TE1 -O3   -152.00  3.00   7_556   1_555   1_555   1_555
PLAT710_ALERT_4_G Delete 1-2-3 or 2-3-4 Linear Torsion Angle ... #         28 Do !
              TE1 -U1  -TE1 -O4    -68.00  3.00   7_556   1_555   1_555   6_555
PLAT710_ALERT_4_G Delete 1-2-3 or 2-3-4 Linear Torsion Angle ... #         39 Do !
              TE1 -U1  -TE1 -O1   -169.00  3.00   7_556   1_555   1_555   1_555
PLAT710_ALERT_4_G Delete 1-2-3 or 2-3-4 Linear Torsion Angle ... #         49 Do !
              TE1 -U1  -TE1 -O2     16.00  3.00   7_556   1_555   1_555   1_555
PLAT710_ALERT_4_G Delete 1-2-3 or 2-3-4 Linear Torsion Angle ... #         60 Do !
              TE1 -U1  -TE1 -O4    109.00  3.00   7_556   1_555   1_555   1_555
PLAT710_ALERT_4_G Delete 1-2-3 or 2-3-4 Linear Torsion Angle ... #         71 Do !
              TE1 -U1  -TE1 -U1      1.00  3.00   7_556   1_555   1_555   4_545
PLAT710_ALERT_4_G Delete 1-2-3 or 2-3-4 Linear Torsion Angle ... #         82 Do !
              TE1 -U1  -TE1 -K1     56.00  3.00   7_556   1_555   1_555   6_556
PLAT710_ALERT_4_G Delete 1-2-3 or 2-3-4 Linear Torsion Angle ... #         93 Do !
              TE1 -U1  -TE1 -K1    112.00  3.00   7_556   1_555   1_555   4_545
PLAT710_ALERT_4_G Delete 1-2-3 or 2-3-4 Linear Torsion Angle ... #        104 Do !
              TE1 -U1  -TE1 -K1    -29.00  3.00   7_556   1_555   1_555   1_555
PLAT710_ALERT_4_G Delete 1-2-3 or 2-3-4 Linear Torsion Angle ... #        115 Do !
              TE1 -U1  -TE1 -K1    -93.00  3.00   7_556   1_555   1_555   7_555
PLAT710_ALERT_4_G Delete 1-2-3 or 2-3-4 Linear Torsion Angle ... #        123 Do !
              O2  -TE1 -O1  -TE1    67.40  1.70   1_555   1_555   1_555   2_555
PLAT710_ALERT_4_G Delete 1-2-3 or 2-3-4 Linear Torsion Angle ... #        134 Do !
              O2  -TE1 -O1  -K1    -84.40  1.80   1_555   1_555   1_555   4_545
PLAT710_ALERT_4_G Delete 1-2-3 or 2-3-4 Linear Torsion Angle ... #        144 Do !
              O2  -TE1 -O1  -K1   -150.70  1.60   1_555   1_555   1_555   3_445
PLAT710_ALERT_4_G Delete 1-2-3 or 2-3-4 Linear Torsion Angle ... #        155 Do !
              O1  -TE1 -O2  -U1    -36.00  1.80   1_555   1_555   1_555   1_555
PLAT710_ALERT_4_G Delete 1-2-3 or 2-3-4 Linear Torsion Angle ... #        165 Do !
              O1  -TE1 -O2  -K1   -147.20  1.70   1_555   1_555   1_555   1_555
PLAT710_ALERT_4_G Delete 1-2-3 or 2-3-4 Linear Torsion Angle ... #        172 Do !
              O5  -TE1 -O2  -K1     -9.70  1.40   1_555   1_555   1_555   2_655
```

```
PLAT710_ALERT_4_G Delete 1-2-3 or 2-3-4 Linear Torsion Angle ... #        173 Do !
              O3  -TE1 -O2  -K1    163.70  1.40   1_555   1_555   1_555   2_655
PLAT710_ALERT_4_G Delete 1-2-3 or 2-3-4 Linear Torsion Angle ... #        174 Do !
              O4  -TE1 -O2  -K1   -105.10  1.40   6_555   1_555   1_555   2_655
PLAT710_ALERT_4_G Delete 1-2-3 or 2-3-4 Linear Torsion Angle ... #        175 Do !
              O1  -TE1 -O2  -K1    119.70  1.80   1_555   1_555   1_555   2_655
PLAT710_ALERT_4_G Delete 1-2-3 or 2-3-4 Linear Torsion Angle ... #        176 Do !
              O4  -TE1 -O2  -K1     71.00  1.40   1_555   1_555   1_555   2_655
PLAT710_ALERT_4_G Delete 1-2-3 or 2-3-4 Linear Torsion Angle ... #        177 Do !
              U1  -TE1 -O2  -K1    155.70  1.50   1_555   1_555   1_555   2_655
PLAT710_ALERT_4_G Delete 1-2-3 or 2-3-4 Linear Torsion Angle ... #        178 Do !
              U1  -TE1 -O2  -K1    -33.20  1.40   4_545   1_555   1_555   2_655
PLAT710_ALERT_4_G Delete 1-2-3 or 2-3-4 Linear Torsion Angle ... #        179 Do !
              K1  -TE1 -O2  -K1     23.50  1.30   6_556   1_555   1_555   2_655
PLAT710_ALERT_4_G Delete 1-2-3 or 2-3-4 Linear Torsion Angle ... #        180 Do !
              K1  -TE1 -O2  -K1     39.30  1.40   4_545   1_555   1_555   2_655
PLAT710_ALERT_4_G Delete 1-2-3 or 2-3-4 Linear Torsion Angle ... #        181 Do !
              K1  -TE1 -O2  -K1    -93.10  1.40   1_555   1_555   1_555   2_655
PLAT710_ALERT_4_G Delete 1-2-3 or 2-3-4 Linear Torsion Angle ... #        182 Do !
              K1  -TE1 -O2  -K1   -141.00  1.40   7_555   1_555   1_555   2_655
PLAT710_ALERT_4_G Delete 1-2-3 or 2-3-4 Linear Torsion Angle ... #        186 Do !
              O1  -TE1 -O2  -K1     96.20  1.70   1_555   1_555   1_555   6_556
PLAT710_ALERT_4_G Delete 1-2-3 or 2-3-4 Linear Torsion Angle ... #        197 Do !
              O2  -U1  -O2  -TE1    46.00  4.00   7_556   1_555   1_555   1_555
PLAT710_ALERT_4_G Delete 1-2-3 or 2-3-4 Linear Torsion Angle ... #        207 Do !
              O2  -U1  -O2  -K1    154.00  4.00   7_556   1_555   1_555   1_555
PLAT710_ALERT_4_G Delete 1-2-3 or 2-3-4 Linear Torsion Angle ... #        218 Do !
              O2  -U1  -O2  -K1   -131.00  4.00   7_556   1_555   1_555   2_655
PLAT710_ALERT_4_G Delete 1-2-3 or 2-3-4 Linear Torsion Angle ... #        228 Do !
              O2  -U1  -O2  -K1    -49.00  4.00   7_556   1_555   1_555   6_556
PLAT710_ALERT_4_G Delete 1-2-3 or 2-3-4 Linear Torsion Angle ... #        278 Do !
              O5  -TE1 -O3  -U1     45.80  1.40   1_555   1_555   1_555   1_555
PLAT710_ALERT_4_G Delete 1-2-3 or 2-3-4 Linear Torsion Angle ... #        288 Do !
              O5  -TE1 -O3  -K1   -103.40  1.30   1_555   1_555   1_555   8_456
PLAT710_ALERT_4_G Delete 1-2-3 or 2-3-4 Linear Torsion Angle ... #        299 Do !
              O5  -TE1 -O3  -K1    146.80  1.30   1_555   1_555   1_555   7_555
PLAT710_ALERT_4_G Delete 1-2-3 or 2-3-4 Linear Torsion Angle ... #        309 Do !
              O3  -U1  -O3  -TE1    25.00 15.00   7_556   1_555   1_555   1_555
PLAT710_ALERT_4_G Delete 1-2-3 or 2-3-4 Linear Torsion Angle ... #        319 Do !
              O3  -U1  -O3  -K1   -168.00 15.00   7_556   1_555   1_555   8_456
PLAT710_ALERT_4_G Delete 1-2-3 or 2-3-4 Linear Torsion Angle ... #        329 Do !
              O3  -U1  -O3  -K1    -74.00 15.00   7_556   1_555   1_555   7_555
PLAT710_ALERT_4_G Delete 1-2-3 or 2-3-4 Linear Torsion Angle ... #        342 Do !
              O4  -TE1 -O4  -TE1   151.00  3.00   6_555   1_555   1_555   6_556
PLAT710_ALERT_4_G Delete 1-2-3 or 2-3-4 Linear Torsion Angle ... #        353 Do !
              O4  -TE1 -O4  -K1    -52.00  3.00   6_555   1_555   1_555   4_545
PLAT710_ALERT_4_G Delete 1-2-3 or 2-3-4 Linear Torsion Angle ... #        363 Do !
              O4  -TE1 -O4  -K1     37.00  3.00   6_555   1_555   1_555   6_556
PLAT710_ALERT_4_G Delete 1-2-3 or 2-3-4 Linear Torsion Angle ... #        371 Do !
              O3  -TE1 -O5  -U1   -170.30  1.10   1_555   1_555   1_555   4_545
PLAT710_ALERT_4_G Delete 1-2-3 or 2-3-4 Linear Torsion Angle ... #        381 Do !
              O3  -TE1 -O5  -K1    -12.70  1.40   1_555   1_555   1_555   6_556
PLAT710_ALERT_4_G Delete 1-2-3 or 2-3-4 Linear Torsion Angle ... #        391 Do !
              O3  -TE1 -O5  -K1     74.70  1.30   1_555   1_555   1_555   4_545
PLAT860_ALERT_3_G Number of Least-Squares Restraints ............          6 Note
PLAT899_ALERT_4_G SHELXL97  is Deprecated and Succeeded by SHELXL-     2019/2 Note
```

```
 0 ALERT level A = Most likely a serious problem - resolve or explain
 0 ALERT level B = A potentially serious problem, consider carefully
 3 ALERT level C = Check. Ensure it is not caused by an omission or oversight
53 ALERT level G = General information/check it is not something unexpected

 4 ALERT type 1 CIF construction/syntax error, inconsistent or missing data
 4 ALERT type 2 Indicator that the structure model may be wrong or deficient
 1 ALERT type 3 Indicator that the structure quality may be low
45 ALERT type 4 Improvement, methodology, query or suggestion
 2 ALERT type 5 Informative message, check
```

It is advisable to attempt to resolve as many as possible of the alerts in all categories. Often the minor alerts point to easily fixed oversights, errors and omissions in your CIF or refinement strategy, so attention to these fine details can be worthwhile. In order to resolve some of the more serious problems it may be necessary to carry out additional measurements or structure refinements. However, the purpose of your study may justify the reported deviations and the more serious of these should normally be commented upon in the discussion or experimental section of a paper or in the "special_details" fields of the CIF. checkCIF was carefully designed to identify outliers and unusual parameters, but every test has its limitations and alerts that are not important in a particular case may appear. Conversely, the absence of alerts does not guarantee there are no aspects of the results needing attention. It is up to the individual to critically assess their own results and, if necessary, seek expert advice.

Publication of your CIF in IUCr journals

A basic structural check has been run on your CIF. These basic checks will be run on all CIFs submitted for publication in IUCr journals (*Acta Crystallographica*, *Journal of Applied Crystallography*, *Journal of Synchrotron Radiation*); however, if you intend to submit to *Acta Crystallographica Section C* or *E* or *IUCrData*, you should make sure that full publication checks are run on the final version of your CIF prior to submission.

Publication of your CIF in other journals

Please refer to the *Notes for Authors* of the relevant journal for any special instructions relating to CIF submission.

PLATON version of 28/11/2022; check.def file version of 28/11/2022

Datablock KUO22Te2O7 - ellipsoid plot

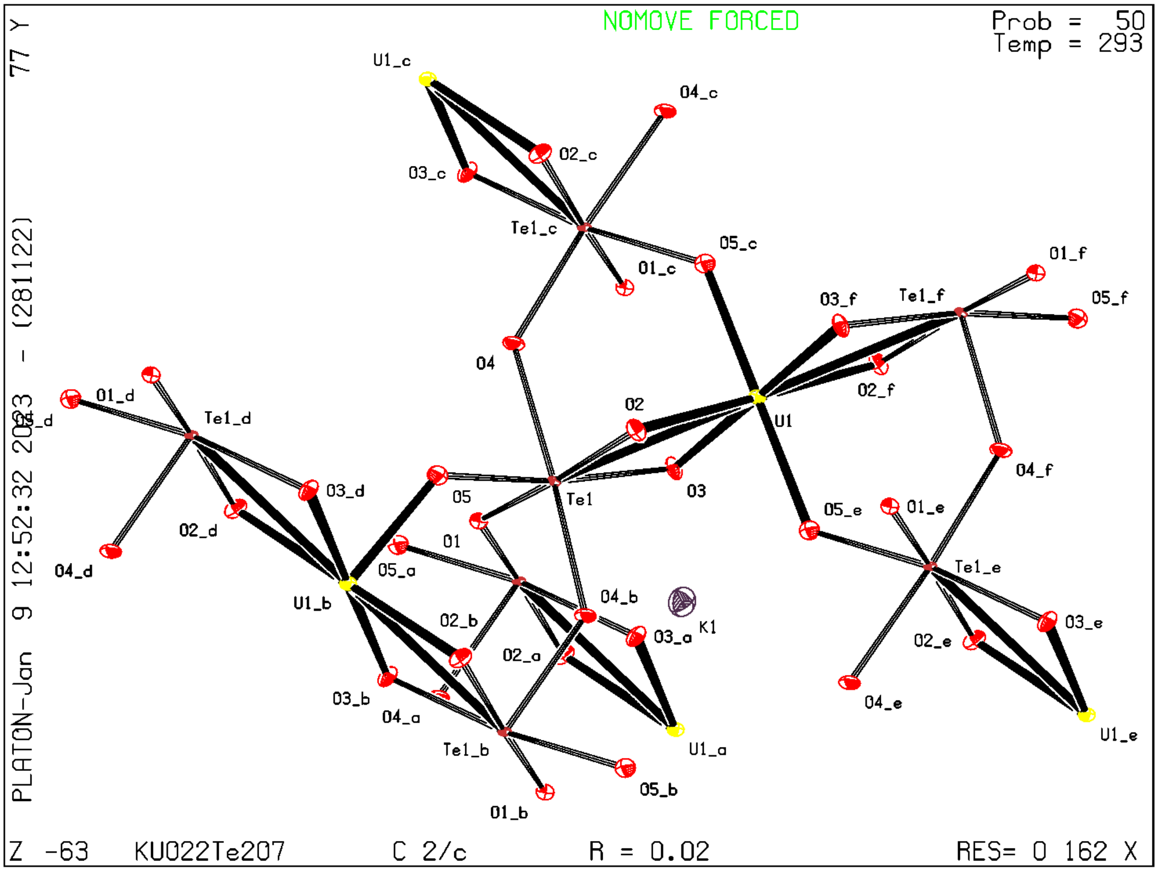

Supplement: Supplementary file 1 [file DataSheet2.zip › CIF and chceckcif/K2UO2Te2O7-checkcif.pdf]
