# Supplementary material for: Understanding of the structural chemistry in the uranium oxo-tellurium system under HT/HP conditions
Source: Front Chem. 2023 Mar 10;11:1152113. doi: 10.3389/fchem.2023.1152113 (PMC10037309; doi:10.3389/fchem.2023.1152113)

checkCIF/PLATON report

Structure factors have been supplied for datablock(s) shelx

THIS REPORT IS FOR GUIDANCE ONLY. IF USED AS PART OF A REVIEW PROCEDURE FOR PUBLICATION, IT SHOULD NOT REPLACE THE EXPERTISE OF AN EXPERIENCED CRYSTALLOGRAPHIC REFEREE.

No syntax errors found. CIF dictionary Interpreting this report

Datablock: shelx

```
Bond precision:   U- O = 0.0057 A          Wavelength=0.71073

Cell:               a=7.5864(4)      b=11.5172(5)      c=7.5143(3)
                    alpha=90         beta=90           gamma=90
Temperature:        293 K

                 Calculated                    Reported
Volume           656.56(5)                     656.56(5)
Space group      C m c a                       C m c a
Hall group       -C 2bc 2                      -C 2bc 2
Moiety formula   Mg O8 Te2 U                   ?
Sum formula      Mg O8 Te2 U                   Mg O8 Te2 U
Mr               645.54                        645.54
Dx,g cm-3        6.531                         6.531
Z                4                             4
Mu (mm-1)        33.535                        33.536
F000             1088.0                        1088.0
F000'            1046.69
h,k,lmax         10,16,10                      10,16,10
Nref             541                           541
Tmin,Tmax        0.075,0.261                   0.854,1.000
Tmin'            0.039

Correction method= # Reported T Limits: Tmin=0.854 Tmax=1.000
AbsCorr = MULTI-SCAN

Data completeness= 1.000           Theta(max)= 30.493

                                                     wR2(reflections)=
R(reflections)= 0.0267( 447)                         0.0662( 541)

S = 1.035                  Npar= 35
```

The following ALERTS were generated. Each ALERT has the format

test-name_ALERT_alert-type_alert-level.

Click on the hyperlinks for more details of the test.

Alert level B

```
PLAT971_ALERT_2_B Check Calcd Resid. Dens.  0.85Ang From U1            2.89 eA-3
```

Alert level C

```
ABSTY02_ALERT_1_C  An _exptl_absorpt_correction_type has been given without
            a literature citation. This should be contained in the
            _exptl_absorpt_process_details field.
            Absorption correction given as multi-scan
PLAT906_ALERT_3_C Large K Value in the Analysis of Variance ......      2.049 Check
PLAT971_ALERT_2_C Check Calcd Resid. Dens.  0.71Ang From U1             2.19 eA-3
PLAT971_ALERT_2_C Check Calcd Resid. Dens.  0.80Ang From Te1            2.19 eA-3
PLAT972_ALERT_2_C Check Calcd Resid. Dens.  1.21Ang From Te1           -1.69 eA-3
PLAT975_ALERT_2_C Check Calcd Resid. Dens.  0.71Ang From O2      .      0.76 eA-3
PLAT975_ALERT_2_C Check Calcd Resid. Dens.  0.86Ang From O3      .      0.59 eA-3
PLAT975_ALERT_2_C Check Calcd Resid. Dens.  0.55Ang From O1      .      0.52 eA-3
PLAT976_ALERT_2_C Check Calcd Resid. Dens.  0.85Ang From O2      .     -0.72 eA-3
PLAT976_ALERT_2_C Check Calcd Resid. Dens.  0.73Ang From O1      .     -0.52 eA-3
```

Alert level G

```
PLAT004_ALERT_5_G Polymeric Structure Found with Maximum Dimension          2 Info
PLAT199_ALERT_1_G Reported _cell_measurement_temperature ..... (K)        293 Check
PLAT200_ALERT_1_G Reported   _diffrn_ambient_temperature ..... (K)        293 Check
PLAT432_ALERT_2_G Short Inter X...Y Contact  Te1      ..O1       .       3.20 Ang.
                                          1-x,1/2+y,3/2-z  =      3_656 Check
PLAT432_ALERT_2_G Short Inter X...Y Contact  Te1      ..O1       .       3.20 Ang.
                                            1/2+x,1/2+y,z  =      5_555 Check
PLAT432_ALERT_2_G Short Inter X...Y Contact  Te1      ..O3       .       3.22 Ang.
                                            x,3/2-y,1/2+z  =     11_576 Check
PLAT432_ALERT_2_G Short Inter X...Y Contact  Te1      ..O3       .       3.22 Ang.
                                          3/2-x,3/2-y,1-z  =     13_666 Check
PLAT794_ALERT_5_G Tentative Bond Valency for U1        (VI)      .       5.90 Info
PLAT794_ALERT_5_G Tentative Bond Valency for Te1       (IV)      .       4.27 Info
PLAT883_ALERT_1_G No Info/Value for _atom_sites_solution_primary .     Please Do !
PLAT910_ALERT_3_G Missing # of FCF Reflection(s) Below Theta(Min).          1 Note
```

0 **ALERT level A** = Most likely a serious problem - resolve or explain
1 **ALERT level B** = A potentially serious problem, consider carefully
10 **ALERT level C** = Check. Ensure it is not caused by an omission or oversight
11 **ALERT level G** = General information/check it is not something unexpected

4 ALERT type 1 CIF construction/syntax error, inconsistent or missing data
13 ALERT type 2 Indicator that the structure model may be wrong or deficient
2 ALERT type 3 Indicator that the structure quality may be low
0 ALERT type 4 Improvement, methodology, query or suggestion
3 ALERT type 5 Informative message, check

It is advisable to attempt to resolve as many as possible of the alerts in all categories. Often the minor alerts point to easily fixed oversights, errors and omissions in your CIF or refinement strategy, so attention to these fine details can be worthwhile. In order to resolve some of the more serious problems it may be necessary to carry out additional measurements or structure refinements. However, the purpose of your study may justify the reported deviations and the more serious of these should normally be commented upon in the discussion or experimental section of a paper or in the "special_details" fields of the CIF. checkCIF was carefully designed to identify outliers and unusual parameters, but every test has its limitations and alerts that are not important in a particular case may appear. Conversely, the absence of alerts does not guarantee there are no aspects of the results needing attention. It is up to the individual to critically assess their own results and, if necessary, seek expert advice.

Publication of your CIF in IUCr journals

A basic structural check has been run on your CIF. These basic checks will be run on all CIFs submitted for publication in IUCr journals (*Acta Crystallographica*, *Journal of Applied Crystallography*, *Journal of Synchrotron Radiation*); however, if you intend to submit to *Acta Crystallographica Section C* or *E* or *IUCrData*, you should make sure that full publication checks are run on the final version of your CIF prior to submission.

Publication of your CIF in other journals

Please refer to the *Notes for Authors* of the relevant journal for any special instructions relating to CIF submission.

PLATON version of 28/11/2022; check.def file version of 28/11/2022

Datablock shelx - ellipsoid plot

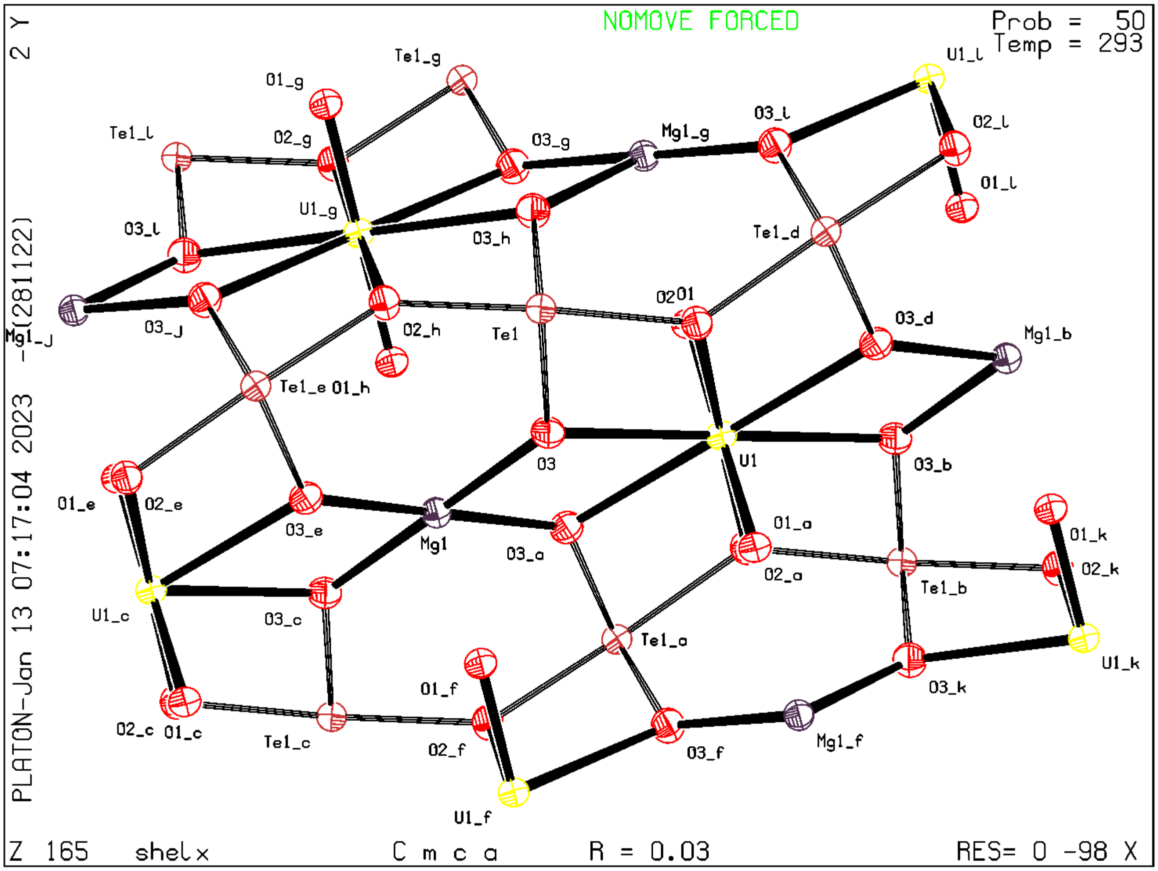

Supplement: Supplementary file 1 [file DataSheet2.zip › CIF and chceckcif/Mg(UO2)(Te2O6)-checkcif.pdf]
