# Supplementary material for: Understanding of the structural chemistry in the uranium oxo-tellurium system under HT/HP conditions
Source: Front Chem. 2023 Mar 10;11:1152113. doi: 10.3389/fchem.2023.1152113 (PMC10037309; doi:10.3389/fchem.2023.1152113)

checkCIF/PLATON report

Structure factors have been supplied for datablock(s) SrUO2TeO32

THIS REPORT IS FOR GUIDANCE ONLY. IF USED AS PART OF A REVIEW PROCEDURE FOR PUBLICATION, IT SHOULD NOT REPLACE THE EXPERTISE OF AN EXPERIENCED CRYSTALLOGRAPHIC REFEREE.

No syntax errors found. CIF dictionary Interpreting this report

Datablock: SrUO2TeO32

```
Bond precision:   U- O = 0.0103 A          Wavelength=0.71073

Cell:              a=11.5299(12)      b=7.8931(9)      c=4.0361(4)
                   alpha=90           beta=90          gamma=90
Temperature:       293 K

                 Calculated                   Reported
Volume           367.31(7)                    367.31(6)
Space group      P b a m                      P b a m
Hall group       -P 2 2ab                     -P 2 2ab
Moiety formula   O8 Te2 U, Sr                 ?
Sum formula      O8 Sr Te2 U                  O8 Sr Te2 U
Mr               708.85                       708.85
Dx,g cm-3        6.409                        6.409
Z                2                            2
Mu (mm-1)        37.070                       37.069
F000             596.0                        596.0
F000'            572.45
h,k,lmax         15,10,5                      14,9,5
Nref             555                          445
Tmin,Tmax        0.080,0.227                  0.854,1.000
Tmin'            0.045

Correction method= # Reported T Limits: Tmin=0.854 Tmax=1.000
AbsCorr = MULTI-SCAN

Data completeness= 0.802           Theta(max)= 28.983

                                                       wR2(reflections)=
R(reflections)= 0.0393( 379)                           0.0990( 445)

S = 1.085                    Npar= 39
```

The following ALERTS were generated. Each ALERT has the format
test-name_ALERT_alert-type_alert-level.
Click on the hyperlinks for more details of the test.

Alert level B

```
PLAT972_ALERT_2_B Check Calcd Resid. Dens.  0.91Ang From U1             -2.52 eA-3
PLAT973_ALERT_2_B Check Calcd Positive Resid. Density on        U1       1.86 eA-3
```

Alert level C

```
ABSTY02_ALERT_1_C  An _exptl_absorpt_correction_type has been given without
            a literature citation. This should be contained in the
            _exptl_absorpt_process_details field.
            Absorption correction given as multi-scan
PLAT911_ALERT_3_C Missing FCF Refl Between Thmin & STh/L=    0.600         20 Report
PLAT971_ALERT_2_C Check Calcd Resid. Dens.  0.97Ang From U1              2.08 eA-3
PLAT971_ALERT_2_C Check Calcd Resid. Dens.  1.20Ang From O4              2.08 eA-3
PLAT971_ALERT_2_C Check Calcd Resid. Dens.  1.00Ang From Te1             1.79 eA-3
PLAT971_ALERT_2_C Check Calcd Resid. Dens.  1.31Ang From O1              1.78 eA-3
PLAT971_ALERT_2_C Check Calcd Resid. Dens.  0.91Ang From Te1             1.73 eA-3
PLAT972_ALERT_2_C Check Calcd Resid. Dens.  0.89Ang From Te1            -2.29 eA-3
PLAT972_ALERT_2_C Check Calcd Resid. Dens.  0.87Ang From Te1            -2.02 eA-3
PLAT972_ALERT_2_C Check Calcd Resid. Dens.  1.50Ang From O3             -2.00 eA-3
PLAT972_ALERT_2_C Check Calcd Resid. Dens.  0.69Ang From Sr1            -1.96 eA-3
PLAT972_ALERT_2_C Check Calcd Resid. Dens.  1.67Ang From O1             -1.82 eA-3
PLAT972_ALERT_2_C Check Calcd Resid. Dens.  1.21Ang From O2             -1.70 eA-3
PLAT972_ALERT_2_C Check Calcd Resid. Dens.  1.22Ang From O4             -1.69 eA-3
PLAT972_ALERT_2_C Check Calcd Resid. Dens.  0.91Ang From Sr1            -1.61 eA-3
PLAT972_ALERT_2_C Check Calcd Resid. Dens.  1.12Ang From Te1            -1.53 eA-3
PLAT972_ALERT_2_C Check Calcd Resid. Dens.  1.12Ang From Te1            -1.53 eA-3
PLAT975_ALERT_2_C Check Calcd Resid. Dens.  0.73Ang From O3      .       1.16 eA-3
PLAT975_ALERT_2_C Check Calcd Resid. Dens.  0.99Ang From O4      .       1.09 eA-3
PLAT975_ALERT_2_C Check Calcd Resid. Dens.  0.61Ang From O3      .       1.08 eA-3
PLAT976_ALERT_2_C Check Calcd Resid. Dens.  0.62Ang From O1      .      -1.16 eA-3
PLAT976_ALERT_2_C Check Calcd Resid. Dens.  0.47Ang From O4      .      -1.08 eA-3
```

Alert level G

```
PLAT004_ALERT_5_G Polymeric Structure Found with Maximum Dimension          1 Info
PLAT199_ALERT_1_G Reported _cell_measurement_temperature ..... (K)        293 Check
PLAT200_ALERT_1_G Reported   _diffrn_ambient_temperature ..... (K)        293 Check
PLAT794_ALERT_5_G Tentative Bond Valency for U1        (VI)      .       6.20 Info
PLAT794_ALERT_5_G Tentative Bond Valency for Te1       (IV)      .       4.01 Info
PLAT883_ALERT_1_G No Info/Value for _atom_sites_solution_primary .     Please Do !
PLAT912_ALERT_4_G Missing # of FCF Reflections Above STh/L=  0.600         71 Note
PLAT913_ALERT_3_G Missing # of Very Strong Reflections in FCF ....          1 Note
PLAT941_ALERT_3_G Average HKL Measurement Multiplicity ...........        1.4 Low
PLAT965_ALERT_2_G The SHELXL WEIGHT Optimisation has not Converged     Please Check
```

0 **ALERT level A** = Most likely a serious problem - resolve or explain
2 **ALERT level B** = A potentially serious problem, consider carefully
22 **ALERT level C** = Check. Ensure it is not caused by an omission or oversight
10 **ALERT level G** = General information/check it is not something unexpected

```
  4 ALERT type 1 CIF construction/syntax error, inconsistent or missing data
 23 ALERT type 2 Indicator that the structure model may be wrong or deficient
  3 ALERT type 3 Indicator that the structure quality may be low
  1 ALERT type 4 Improvement, methodology, query or suggestion
  3 ALERT type 5 Informative message, check
```

It is advisable to attempt to resolve as many as possible of the alerts in all categories. Often the minor alerts point to easily fixed oversights, errors and omissions in your CIF or refinement strategy, so attention to these fine details can be worthwhile. In order to resolve some of the more serious problems it may be necessary to carry out additional measurements or structure refinements. However, the purpose of your study may justify the reported deviations and the more serious of these should normally be commented upon in the discussion or experimental section of a paper or in the "special_details" fields of the CIF. checkCIF was carefully designed to identify outliers and unusual parameters, but every test has its limitations and alerts that are not important in a particular case may appear. Conversely, the absence of alerts does not guarantee there are no aspects of the results needing attention. It is up to the individual to critically assess their own results and, if necessary, seek expert advice.

Publication of your CIF in IUCr journals

A basic structural check has been run on your CIF. These basic checks will be run on all CIFs submitted for publication in IUCr journals (*Acta Crystallographica*, *Journal of Applied Crystallography*, *Journal of Synchrotron Radiation*); however, if you intend to submit to *Acta Crystallographica Section C* or *E* or *IUCrData*, you should make sure that full publication checks are run on the final version of your CIF prior to submission.

Publication of your CIF in other journals

Please refer to the *Notes for Authors* of the relevant journal for any special instructions relating to CIF submission.

PLATON version of 28/11/2022; check.def file version of 28/11/2022

Datablock SrUO2TeO32 - ellipsoid plot

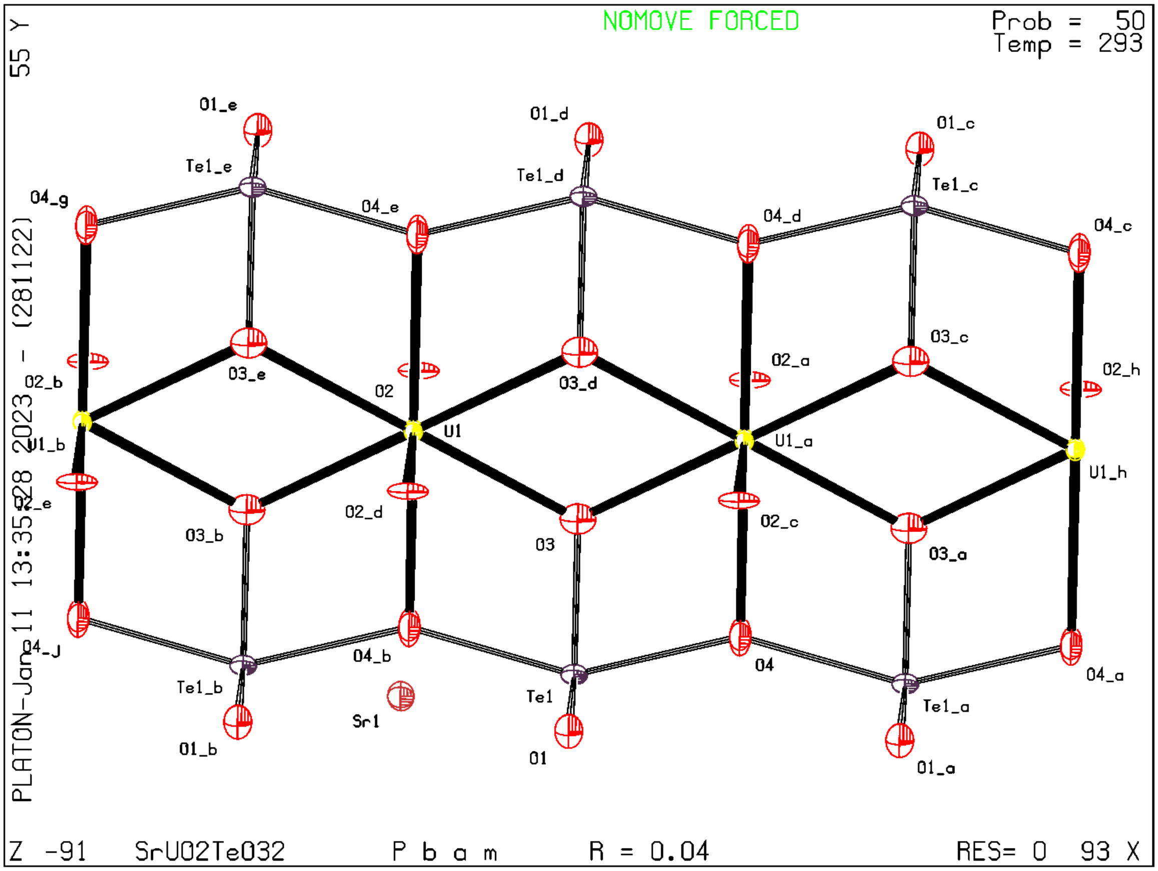

Supplement: Supplementary file 1 [file DataSheet2.zip › CIF and chceckcif/Sr(UO2)(TeO3)2-checkcif.pdf]
