# Supplementary material for: Understanding of the structural chemistry in the uranium oxo-tellurium system under HT/HP conditions
Source: Front Chem. 2023 Mar 10;11:1152113. doi: 10.3389/fchem.2023.1152113 (PMC10037309; doi:10.3389/fchem.2023.1152113)

checkCIF/PLATON report

Structure factors have been supplied for datablock(s) SrUO2TeO5

THIS REPORT IS FOR GUIDANCE ONLY. IF USED AS PART OF A REVIEW PROCEDURE FOR PUBLICATION, IT SHOULD NOT REPLACE THE EXPERTISE OF AN EXPERIENCED CRYSTALLOGRAPHIC REFEREE.

No syntax errors found.   CIF dictionary   Interpreting this report

Datablock: SrUO2TeO5

```
Bond precision:   U- O = 0.0061 A          Wavelength=0.71073

Cell:              a=12.5377(2)      b=12.4599(2)      c=7.57807(16)
                   alpha=90          beta=90           gamma=90
Temperature:       293 K

                Calculated                   Reported
Volume          1183.84(4)                   1183.83(4)
Space group     P b a m                      P b a m
Hall group      -P 2 2ab                     -P 2 2ab
Moiety formula  O28 Te4 U4, 4(Sr)            ?
Sum formula     O28 Sr4 Te4 U4               O7 Sr Te U
Mr              2261.00                      565.25
Dx,g cm-3       6.343                        6.343
Z               2                            8
Mu (mm-1)       41.146                       41.146
F000            1904.0                       1904.0
F000'           1814.04
h,k,lmax        16,15,9                      16,15,9
Nref            1403                         1403
Tmin,Tmax                                    0.850,1.000
Tmin'

Correction method= # Reported T Limits: Tmin=0.850 Tmax=1.000
AbsCorr = MULTI-SCAN

Data completeness= 1.000            Theta(max)= 27.096

R(reflections)= 0.0214( 1345)                    wR2(reflections)=
                                                 0.0589( 1403)
S = 1.093                   Npar= 112
```

The following ALERTS were generated. Each ALERT has the format
test-name_ALERT_alert-type_alert-level.
Click on the hyperlinks for more details of the test.

Alert level B

```
PLAT972_ALERT_2_B Check Calcd Resid. Dens.  0.36Ang From Sr2          -2.74 eA-3
PLAT972_ALERT_2_B Check Calcd Resid. Dens.  0.53Ang From Sr1          -2.61 eA-3
PLAT972_ALERT_2_B Check Calcd Resid. Dens.  0.53Ang From Sr1          -2.61 eA-3
PLAT972_ALERT_2_B Check Calcd Resid. Dens.  0.37Ang From Sr1          -2.53 eA-3
```

Alert level C

```
ABSTY02_ALERT_1_C  An _exptl_absorpt_correction_type has been given without
            a literature citation. This should be contained in the
            _exptl_absorpt_process_details field.
            Absorption correction given as multi-scan
PLAT053_ALERT_1_C Minimum Crystal Dimension Missing (or Error) ...     Please Check
PLAT054_ALERT_1_C Medium  Crystal Dimension Missing (or Error) ...     Please Check
PLAT055_ALERT_1_C Maximum Crystal Dimension Missing (or Error) ...     Please Check
PLAT972_ALERT_2_C Check Calcd Resid. Dens.  0.41Ang From Sr1          -2.50 eA-3
PLAT972_ALERT_2_C Check Calcd Resid. Dens.  0.49Ang From Sr2          -2.31 eA-3
PLAT975_ALERT_2_C Check Calcd Resid. Dens.  1.04Ang From O4      .     0.79 eA-3
PLAT975_ALERT_2_C Check Calcd Resid. Dens.  0.62Ang From O10     .     0.63 eA-3
```

Alert level G

```
PLAT004_ALERT_5_G Polymeric Structure Found with Maximum Dimension         3 Info
PLAT045_ALERT_1_G Calculated and Reported Z Differ by a Factor ...     0.250 Check
PLAT083_ALERT_2_G SHELXL Second Parameter in WGHT  Unusually Large     19.14 Why ?
PLAT199_ALERT_1_G Reported _cell_measurement_temperature ..... (K)       293 Check
PLAT200_ALERT_1_G Reported   _diffrn_ambient_temperature ..... (K)       293 Check
PLAT300_ALERT_4_G Atom Site Occupancy of Sr2        Constrained at    0.6667 Check
PLAT300_ALERT_4_G Atom Site Occupancy of Sr3        Constrained at    0.3333 Check
PLAT302_ALERT_4_G Anion/Solvent/Minor-Residue Disorder (Resd  3  )      100% Note
PLAT302_ALERT_4_G Anion/Solvent/Minor-Residue Disorder (Resd  4  )      100% Note
PLAT794_ALERT_5_G Tentative Bond Valency for U1        (VI)      .      5.99 Info
PLAT794_ALERT_5_G Tentative Bond Valency for U2        (VI)      .      5.95 Info
PLAT794_ALERT_5_G Tentative Bond Valency for Te1       (VI)      .      6.06 Info
PLAT883_ALERT_1_G No Info/Value for _atom_sites_solution_primary .     Please Do !
PLAT910_ALERT_3_G Missing # of FCF Reflection(s) Below Theta(Min).         1 Note
PLAT965_ALERT_2_G The SHELXL WEIGHT Optimisation has not Converged     Please Check
```

0 **ALERT level A** = Most likely a serious problem - resolve or explain
4 **ALERT level B** = A potentially serious problem, consider carefully
8 **ALERT level C** = Check. Ensure it is not caused by an omission or oversight
15 **ALERT level G** = General information/check it is not something unexpected

8 ALERT type 1 CIF construction/syntax error, inconsistent or missing data
10 ALERT type 2 Indicator that the structure model may be wrong or deficient
1 ALERT type 3 Indicator that the structure quality may be low
4 ALERT type 4 Improvement, methodology, query or suggestion
4 ALERT type 5 Informative message, check

It is advisable to attempt to resolve as many as possible of the alerts in all categories. Often the minor alerts point to easily fixed oversights, errors and omissions in your CIF or refinement strategy, so attention to these fine details can be worthwhile. In order to resolve some of the more serious problems it may be necessary to carry out additional measurements or structure refinements. However, the purpose of your study may justify the reported deviations and the more serious of these should normally be commented upon in the discussion or experimental section of a paper or in the "special_details" fields of the CIF. checkCIF was carefully designed to identify outliers and unusual parameters, but every test has its limitations and alerts that are not important in a particular case may appear. Conversely, the absence of alerts does not guarantee there are no aspects of the results needing attention. It is up to the individual to critically assess their own results and, if necessary, seek expert advice.

Publication of your CIF in IUCr journals

A basic structural check has been run on your CIF. These basic checks will be run on all CIFs submitted for publication in IUCr journals (*Acta Crystallographica*, *Journal of Applied Crystallography*, *Journal of Synchrotron Radiation*); however, if you intend to submit to *Acta Crystallographica Section C* or *E* or *IUCrData*, you should make sure that full publication checks are run on the final version of your CIF prior to submission.

Publication of your CIF in other journals

Please refer to the *Notes for Authors* of the relevant journal for any special instructions relating to CIF submission.

PLATON version of 28/11/2022; check.def file version of 28/11/2022

Datablock SrUO2TeO5 - ellipsoid plot

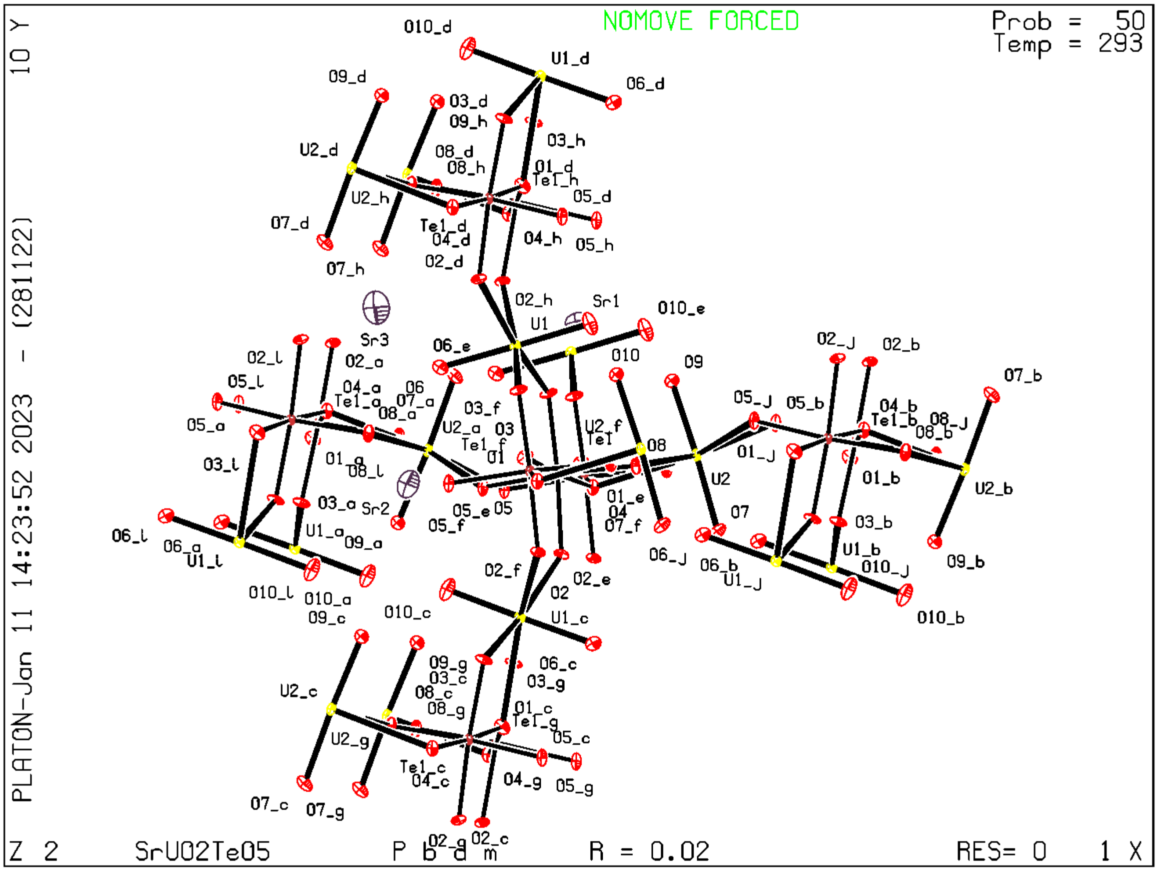

Supplement: Supplementary file 1 [file DataSheet2.zip › CIF and chceckcif/SrUO2(TeO5)-checkcif.pdf]
