# Supplementary material for: Understanding of the structural chemistry in the uranium oxo-tellurium system under HT/HP conditions
Source: Front Chem. 2023 Mar 10;11:1152113. doi: 10.3389/fchem.2023.1152113 (PMC10037309; doi:10.3389/fchem.2023.1152113)
Supplement: Supplementary file 2 [file DataSheet1.docx]

Supporting Information

**Table S1. Important Bond Lengths (angstroms) and Important Bond angles (degrees) for**  **K_2_(UO_2_)Te_2_O_7_, Mg(UO_2_)(Te_2_O_6_)，Sr[(UO_2_)(TeO_3_)_2_], Sr_2_[(UO_2_)_2_(TeO_5_)_2_]**

| **K_2_(UO_2_)Te_2_O_7_** | | **Mg(UO_2_)(Te_2_O_6_)** | |
| --- | --- | --- | --- |
| U(1)-O(3) | 1.992(4) | U(1)-O(1) | 1.805(6) |
| U(1)-O(3) | 1.992(4) | U(1)-O(1) | 1.805(6) |
| U(1)-O(5) | 1.995(4) | U(1)-O(2) | 2.361(7) |
| U(1)-O(5) | 1.995(4) | U(1)-O(2) | 2.361(7) |
| U(1)-O(2) | 2.031(4) | U(1)-O(3) | 2.527(5) |
| U(1)-O(2) | 2.031(4) | U(1)-O(3) | 2.527(5) |
| Te(1)-O(5) | 1.888(4) | U(1)-O(3) | 2.527(5) |
| Te(1)-O(3) | 1.909(4) | U(1)-O(3) | 2.527(5) |
| Te(1)-O(4) | 1.924(4) | Te(1)-O(3) | 1.881(4) |
| Te(1)-O(1) | 1.947(3) | Te(1)-O(3) | 1.881(4) |
| Te(1)-O(2) | 1.958(4) | Te(1)-O(2) | 2.042(3) |
| Te(1)-O(4) | 1.979(4) | Te(1)-O(2) | 2.042(3) |
| K(1)-O(3) | 2.676(5) | Mg(1)-O(3) | 2.051(5) |
| K(1)-O(4) | 2.688(5) | Mg(1)-O(3) | 2.051(5) |
| K(1)-O(5) | 2.709(5) | Mg(1)-O(3) | 2.051(5) |
| K(1)-O(2) | 2.911(5) | Mg(1)-O(3) | 2.051(5) |
| K(1)-O(4) | 2.942(4) | Mg(1)-O(1) | 2.284(6) |
| K(1)-O(2) | 2.982(5) | Mg(1)-O(1) | 2.284(6) |
| K(1)-O(5) | 3.016(5) |  |  |
| K(1)-O(1) | 3.099(5) | O(1)-U(1)-O(1) | 180.0 |
| K(1)-O(2) | 3.138(5) | O(1)-U(1)-O(2) | 94.9(3) |
| K(1)-O(3) | 3.304(5) | O(1)-U(1)-O(2) | 85.1(3) |
|  |  | O(1)-U(1)-O(2) | 85.1(3) |
| O(3)-U(1)-O(3) | 179.999(2) | O(1)-U(1)-O(2) | 94.9(3) |
| O(3)-U(1)-O(5) | 94.44(18) | O(2)-U(1)-O(2) | 180.00(17) |
| O(3)-U(1)-O(5) | 85.56(18) | O(1)-U(1)-O(3) | 87.64(16) |
| O(3)-U(1)-O(5) | 85.56(18) | O(1)-U(1)-O(3) | 92.36(16) |
| O(3)-U(1)-O(5) | 94.44(18) | O(2)-U(1)-O(3) | 60.25(10) |
| O(5)-U(1)-O(5) | 180.0 | O(2)-U(1)-O(3) | 119.75(11) |
| O(3)-U(1)-O(2) | 78.66(17) | O(1)-U(1)-O(3) | 92.36(16) |
| O(3)-U(1)-O(2) | 101.35(17) | O(1)-U(1)-O(3) | 87.64(16) |
| O(5)-U(1)-O(2) | 92.63(18) | O(2)-U(1)-O(3) | 119.75(11) |
| O(5)-U(1)-O(2) | 87.38(18) | O(2)-U(1)-O(3) | 60.25(10) |
| O(3)-U(1)-O(2) | 101.35(17) | O(3)-U(1)-O(3) | 180.0 |
| O(3)-U(1)-O(2) | 78.65(17) | O(1)-U(1)-O(3) | 87.64(16) |
| O(5)-U(1)-O(2) | 87.38(18) | O(1)-U(1)-O(3) | 92.36(16) |
| O(5)-U(1)-O(2) | 92.62(18) | O(2)-U(1)-O(3) | 60.25(10) |
| O(2)-U(1)-O(2) | 180.0 | O(2)-U(1)-O(3) | 119.75(11) |
| O(5)-Te(1)-O(3) | 171.94(19) | O(3)-U(1)-O(3) | 119.6(2) |
| O(5)-Te(1)-O(4) | 95.23(19) | O(3)-U(1)-O(3) | 60.4(2) |
| O(3)-Te(1)-O(4) | 91.41(19) | O(1)-U(1)-O(3) | 92.36(16) |
| O(5)-Te(1)-O(1) | 91.3(2) | O(1)-U(1)-O(3) | 87.64(16) |
| O(3)-Te(1)-O(1) | 92.88(19) | O(2)-U(1)-O(3) | 119.75(11) |
| O(4)-Te(1)-O(1) | 92.93(14) | O(2)-U(1)-O(3) | 60.25(10) |
| O(5)-Te(1)-O(2) | 92.73(19) | O(3)-U(1)-O(3) | 60.4(2) |
| O(3)-Te(1)-O(2) | 82.50(18) | O(3)-U(1)-O(3) | 119.6(2) |
| O(4)-Te(1)-O(2) | 91.61(18) | O(3)-U(1)-O(3) | 180.0 |
| O(1)-Te(1)-O(2) | 173.60(14) | O(3)-Te(1)-O(3) | 99.8(3) |
| O(5)-Te(1)-O(4) | 80.83(19) | O(3)-Te(1)-O(2) | 77.5(2) |
| O(3)-Te(1)-O(4) | 92.50(19) | O(3)-Te(1)-O(2) | 88.9(2) |
| O(4)-Te(1)-O(4) | 176.04(15) | O(3)-Te(1)-O(2) | 88.9(2) |
| O(1)-Te(1)-O(4) | 87.48(14) | O(3)-Te(1)-O(2) | 77.5(2) |
| O(2)-Te(1)-O(4) | 88.29(18) | O(2)-Te(1)-O(2) | 158.9(4) |
| O(3)-K(1)-O(4) | 69.25(13) | O(3)-Te(1)-O(1) | 83.21(15) |
| O(3)-K(1)-O(5) | 105.99(14) | O(3)-Te(1)-O(1) | 42.11(15) |
| O(4)-K(1)-O(5) | 74.34(13) | O(2)-Te(1)-O(1) | 122.49(19) |
| O(3)-K(1)-O(2) | 142.18(14) | O(2)-Te(1)-O(1) | 38.53(18) |
| O(4)-K(1)-O(2) | 107.68(13) | O(3)-Te(1)-O(1) | 42.11(15) |
| O(5)-K(1)-O(2) | 109.27(14) | O(3)-Te(1)-O(1) | 83.21(15) |
| O(3)-K(1)-O(4) | 155.70(13) | O(2)-Te(1)-O(1) | 38.54(18) |
| O(4)-K(1)-O(4) | 91.77(14) | O(2)-Te(1)-O(1) | 122.49(19) |
| O(5)-K(1)-O(4) | 52.49(13) | O(3)-Mg(1)-O(3) | 103.4(3) |
| O(2)-K(1)-O(4) | 56.78(12) | O(3)-Mg(1)-O(3) | 76.6(3) |
| O(3)-K(1)-O(2) | 66.49(12) | O(3)-Mg(1)-O(3) | 180.0 |
| O(4)-K(1)-O(2) | 129.63(14) | O(3)-Mg(1)-O(3) | 180.0 |
| O(5)-K(1)-O(2) | 140.65(14) | O(3)-Mg(1)-O(3) | 76.6(3) |
| O(2)-K(1)-O(2) | 93.40(13) | O(3)-Mg(1)-O(3) | 103.4(3) |
| O(4)-K(1)-O(2) | 136.59(13) | O(3)-Mg(1)-O(1) | 89.32(17) |
| O(3)-K(1)-O(5) | 109.01(14) | O(3)-Mg(1)-O(1) | 89.32(18) |
| O(4)-K(1)-O(5) | 51.77(12) | O(3)-Mg(1)-O(1) | 90.68(18) |
| O(5)-K(1)-O(5) | 93.66(13) | O(3)-Mg(1)-O(1) | 90.68(17) |
| O(2)-K(1)-O(5) | 55.94(12) | O(3)-Mg(1)-O(1) | 90.68(17) |
| O(4)-K(1)-O(5) | 66.37(12) | O(3)-Mg(1)-O(1) | 90.68(18) |
| O(2)-K(1)-O(5) | 125.62(13) | O(3)-Mg(1)-O(1) | 89.32(18) |
| O(3)-K(1)-O(1) | 62.56(10) | O(3)-Mg(1)-O(1) | 89.32(17) |
| O(4)-K(1)-O(1) | 55.39(11) | O(1)-Mg(1)-O(1) | 180.0 |
| O(5)-K(1)-O(1) | 129.54(12) |  |  |
| O(2)-K(1)-O(1) | 84.28(11) |  |  |
| O(4)-K(1)-O(1) | 119.50(10) |  |  |
| O(2)-K(1)-O(1) | 83.11(11) |  |  |
| O(5)-K(1)-O(1) | 53.28(9) |  |  |
| O(3)-K(1)-O(2) | 128.27(13) |  |  |
| O(4)-K(1)-O(2) | 130.02(13) |  |  |
| O(5)-K(1)-O(2) | 56.28(12) |  |  |
| O(2)-K(1)-O(2) | 83.91(9) |  |  |
| O(4)-K(1)-O(2) | 53.50(12) |  |  |
| O(2)-K(1)-O(2) | 96.62(12) |  |  |
| O(5)-K(1)-O(2) | 119.45(12) |  |  |
| O(1)-K(1)-O(2) | 168.05(11) |  |  |
| O(3)-K(1)-O(3) | 53.99(15) |  |  |
| O(4)-K(1)-O(3) | 53.29(12) |  |  |
| O(5)-K(1)-O(3) | 52.40(12) |  |  |
| O(2)-K(1)-O(3) | 154.15(13) |  |  |
| O(4)-K(1)-O(3) | 102.71(12) |  |  |
| O(2)-K(1)-O(3) | 112.21(12) |  |  |
| O(5)-K(1)-O(3) | 103.29(12) |  |  |
| O(1)-K(1)-O(3) | 94.94(10) |  |  |
| O(2)-K(1)-O(3) | 96.20(12) |  |  |
| **Sr[(UO_2_)(TeO_3_)_2_]** | | **Sr_2_[(UO_2_)_2_(TeO_5_)_2_]** | |
| U(1)-O(2) | 1.825(13) | U(1)-O(10) | 1.815(8) |
| U(1)-O(2) | 1.825(13) | U(1)-O(6) | 1.816(7) |
| U(1)-O(3) | 2.349(7) | U(1)-O(2) | 2.280(5) |
| U(1)-O(3) | 2.349(7) | U(1)-O(2) | 2.280(5) |
| U(1)-O(3) | 2.349(7) | U(1)-O(3) | 2.326(5) |
| U(1)-O(3) | 2.349(7) | U(1)-O(3) | 2.326(5) |
| U(1)-O(4) | 2.483(14) | U(1)-O(1) | 2.561(7) |
| U(1)-O(4) | 2.483(14) | U(2)-O(9) | 1.810(7) |
| Sr(1)-O(1) | 2.525(7) | U(2)-O(7) | 1.822(7) |
| Sr(1)-O(1) | 2.525(7) | U(2)-O(5) | 2.295(5) |
| Sr(1)-O(1) | 2.525(7) | U(2)-O(5) | 2.295(5) |
| Sr(1)-O(1) | 2.525(7) | U(2)-O(8) | 2.336(5) |
| Sr(1)-O(2) | 2.569(13) | U(2)-O(8) | 2.336(5) |
| Sr(1)-O(2) | 2.569(13) | U(2)-O(4) | 2.525(7) |
| Sr(1)-O(4) | 3.029(14) | Te(1)-O(3) | 1.880(5) |
| Sr(1)-O(4) | 3.029(14) | Te(1)-O(8) | 1.881(5) |
| Te(1)-O(1) | 1.849(13) | Te(1)-O(2) | 1.889(9) |
| Te(1)-O(3) | 1.919(13) | Te(1)-O(5) | 1.891(5) |
| Te(1)-O(4) | 2.102(4) | Te(1)-O(1) | 1.975(2) |
| Te(1)-O(4) | 2.102(4) | Te(1)-O(4) | 1.980(2) |
|  |  | Sr(1)-O(3) | 2.460(5) |
| O(2)-U(1)-O(2) | 180.0 | Sr(1)-O(3) | 2.460(5) |
| O(2)-U(1)-O(3) | 94.3(4) | Sr(1)-O(9) | 2.606(7) |
| O(2)-U(1)-O(3) | 85.7(4) | Sr(1)-O(2) | 2.728(5) |
| O(2)-U(1)-O(3) | 85.7(4) | Sr(1)-O(2) | 2.728(5) |
| O(2)-U(1)-O(3) | 94.3(4) | Sr(1)-O(5) | 2.813(5) |
| O(3)-U(1)-O(3) | 180.0 | Sr(1)-O(5) | 2.813(5) |
| O(2)-U(1)-O(3) | 85.7(4) | Sr(1)-O(4) | 2.963(7) |
| O(2)-U(1)-O(3) | 94.3(4) | Sr(1)-O(7) | 3.110(7) |
| O(3)-U(1)-O(3) | 61.6(5) | Sr(1)-O(9) | 3.175(7) |
| O(3)-U(1)-O(3) | 118.4(5) | Sr(2)-O(8) | 2.429(5) |
| O(2)-U(1)-O(3) | 94.3(4) | Sr(2)-O(8) | 2.429(5) |
| O(2)-U(1)-O(3) | 85.7(4) | Sr(2)-O(10) | 2.595(8) |
| O(3)-U(1)-O(3) | 118.4(5) | Sr(2)-O(5) | 2.689(5) |
| O(3)-U(1)-O(3) | 61.6(5) | Sr(2)-O(5) | 2.689(5) |
| O(3)-U(1)-O(3) | 180.0(7) | Sr(2)-O(6) | 2.790(7) |
| O(2)-U(1)-O(4) | 98.6(5) | Sr(2)-O(1) | 2.797(8) |
| O(2)-U(1)-O(4) | 81.4(5) | Sr(2)-O(10) | 2.985(9) |
| O(3)-U(1)-O(4) | 119.3(3) | Sr(3)-O(2) | 2.454(5) |
| O(3)-U(1)-O(4) | 60.7(3) | Sr(3)-O(2) | 2.454(5) |
| O(3)-U(1)-O(4) | 60.7(3) | Sr(3)-O(6) | 2.563(6) |
| O(3)-U(1)-O(4) | 119.3(3) | Sr(3)-O(6) | 2.563(6) |
| O(2)-U(1)-O(4) | 81.4(5) | Sr(3)-O(7) | 2.590(6) |
| O(2)-U(1)-O(4) | 98.6(5) | Sr(3)-O(7) | 2.590(7) |
| O(3)-U(1)-O(4) | 60.7(3) | Sr(3)-O(8) | 2.935(5) |
| O(3)-U(1)-O(4) | 119.3(3) | Sr(3)-O(8) | 2.935(5) |
| O(3)-U(1)-O(4) | 119.3(3) |  |  |
| O(3)-U(1)-O(4) | 60.7(3) | O(10)-U(1)-O(6) | 179.0(3) |
| O(4)-U(1)-O(4) | 180.0 | O(10)-U(1)-O(2) | 93.2(2) |
| O(1)-Sr(1)-O(1) | 106.1(4) | O(6)-U(1)-O(2) | 86.1(2) |
| O(1)-Sr(1)-O(1) | 73.9(4) | O(10)-U(1)-O(2) | 93.2(2) |
| O(1)-Sr(1)-O(1) | 180.0(5) | O(6)-U(1)-O(2) | 86.1(2) |
| O(1)-Sr(1)-O(1) | 180.0 | O(2)-U(1)-O(2) | 89.1(3) |
| O(1)-Sr(1)-O(1) | 73.9(4) | O(10)-U(1)-O(3) | 87.32(18) |
| O(1)-Sr(1)-O(1) | 106.1(4) | O(6)-U(1)-O(3) | 93.15(17) |
| O(1)-Sr(1)-O(2) | 89.4(4) | O(2)-U(1)-O(3) | 74.52(18) |
| O(1)-Sr(1)-O(2) | 89.4(4) | O(2)-U(1)-O(3) | 163.62(17) |
| O(1)-Sr(1)-O(2) | 90.6(4) | O(10)-U(1)-O(3) | 87.32(18) |
| O(1)-Sr(1)-O(2) | 90.6(4) | O(6)-U(1)-O(3) | 93.14(17) |
| O(1)-Sr(1)-O(2) | 90.6(4) | O(2)-U(1)-O(3) | 163.62(17) |
| O(1)-Sr(1)-O(2) | 90.6(4) | O(2)-U(1)-O(3) | 74.51(18) |
| O(1)-Sr(1)-O(2) | 89.4(4) | O(3)-U(1)-O(3) | 121.9(2) |
| O(1)-Sr(1)-O(2) | 89.4(4) | O(10)-U(1)-O(1) | 101.3(3) |
| O(2)-Sr(1)-O(2) | 180.0 | O(6)-U(1)-O(1) | 79.7(3) |
| O(1)-Sr(1)-O(4) | 58.7(3) | O(2)-U(1)-O(1) | 133.30(13) |
| O(1)-Sr(1)-O(4) | 58.7(3) | O(2)-U(1)-O(1) | 133.30(13) |
| O(1)-Sr(1)-O(4) | 121.3(3) | O(3)-U(1)-O(1) | 62.27(13) |
| O(1)-Sr(1)-O(4) | 121.3(3) | O(3)-U(1)-O(1) | 62.27(13) |
| O(2)-Sr(1)-O(4) | 119.4(4) | O(9)-U(2)-O(7) | 177.3(3) |
| O(2)-Sr(1)-O(4) | 60.6(4) | O(9)-U(2)-O(5) | 84.5(2) |
| O(1)-Sr(1)-O(4) | 121.3(3) | O(7)-U(2)-O(5) | 97.6 (2) |
| O(1)-Sr(1)-O(4) | 121.3(3) | O(9)-U(2)-O(5) | 84.5(2) |
| O(1)-Sr(1)-O(4) | 58.7(3) | O(7)-U(2)-O(5) | 97.6 (2) |
| O(1)-Sr(1)-O(4) | 58.7(3) | O(5)-U(2)-O(5) | 79.0(2) |
| O(2)-Sr(1)-O(4) | 60.6(4) | O(9)-U(2)-O(8) | 88.17(16) |
| O(2)-Sr(1)-O(4) | 119.4(4) | O(7)-U(2)-O(8) | 90.58(17) |
| O(4)-Sr(1)-O(4) | 180.0 | O(5)-U(2)-O(8) | 77.86(18) |
| O(1)-Te(1)-O(3) | 103.4(6) | O(5)-U(2)-O(8) | 156.30(18) |
| O(1)-Te(1)-O(4) | 88.3(4) | O(9)-U(2)-O(8) | 88.17(16) |
| O(3)-Te(1)-O(4) | 74.7(4) | O(7)-U(2)-O(8) | 90.58(17) |
| O(1)-Te(1)-O(4) | 88.3(4) | O(5)-U(2)-O(8) | 156.30(18) |
| O(3)-Te(1)-O(4) | 74.7(4) | O(5)-U(2)-O(8) | 77.86(18) |
| O(4)-Te(1)-O(4) | 147.5(7) | O(8)-U(2)-O(8) | 124.5(3) |
|  |  | O(9)-U(2)-O(4) | 88.3(3) |
|  |  | O(7)-U(2)-O(4) | 89.0(3) |
|  |  | O(5)-U(2)-O(4) | 139.68(13) |
|  |  | O(5)-U(2)-O(4) | 139.68(13) |
|  |  | O(8)-U(2)-O(4) | 62.26(13) |
|  |  | O(8)-U(2)-O(4) | 62.26(13) |
|  |  | O(3)-Te(1)-O(8) | 92.1(2) |
|  |  | O(3)-Te(1)-O(2) | 174.5(2) |
|  |  | O(8)-Te(1)-O(2) | 90.4(2) |
|  |  | O(3)-Te(1)-O(5) | 90.8(2) |
|  |  | O(8)-Te(1)-O(5) | 171.2(2) |
|  |  | O(2)-Te(1)-O(5) | 87.5(2) |
|  |  | O(3)-Te(1)-O(1) | 82.2(3) |
|  |  | O(8)-Te(1)-O(1) | 96.4(3) |
|  |  | O(2)-Te(1)-O(1) | 92.6(3) |
|  |  | O(5)-Te(1)-O(1) | 92.2(3) |
|  |  | O(3)-Te(1)-O(4) | 95.7(3) |
|  |  | O(8)-Te(1)-O(4) | 81.4(3) |
|  |  | O(2)-Te(1)-O(4) | 89.6(3) |
|  |  | O(5)-Te(1)-O(4) | 90.1(3) |
|  |  | O(1)-Te(1)-O(4) | 176.9(3) |
|  |  | O(3)-Sr(1)-O(3) | 91.1(2) |
|  |  | O(3)-Sr(1)-O(9) | 76.51(16) |
|  |  | O(3)-Sr(1)-O(9) | 76.51(16) |
|  |  | O(3)-Sr(1)-O(2) | 136.05(17) |
|  |  | O(3)-Sr(1)-O(2) | 64.79(15) |
|  |  | O(9)-Sr(1)-O(2) | 126.60(11) |
|  |  | O(3)-Sr(1)-O(2) | 64.79(15) |
|  |  | O(3)-Sr(1)-O(2) | 136.05(17) |
|  |  | O(9)-Sr(1)-O(2) | 126.60(11) |
|  |  | O(2)-Sr(1)-O(2) | 106.8(2) |
|  |  | O(3)-Sr(1)-O(5) | 103.06(16) |
|  |  | O(3)-Sr(1)-O(5) | 165.25(16) |
|  |  | O(9)-Sr(1)-O(5) | 102.51(17) |
|  |  | O(2)-Sr(1)-O(5) | 106.12(14) |
|  |  | O(2)-Sr(1)-O(5) | 56.24(14) |
|  |  | O(3)-Sr(1)-O(5) | 165.25(16) |
|  |  | O(3)-Sr(1)-O(5) | 103.06(16) |
|  |  | O(9)-Sr(1)-O(5) | 102.51(17) |
|  |  | O(2)-Sr(1)-O(5) | 56.24(14) |
|  |  | O(2)-Sr(1)-O(5) | 106.12(14) |
|  |  | O(5)-Sr(1)-O(5) | 62.5(2) |
|  |  | O(3)-Sr(1)-O(4) | 119.65(14) |
|  |  | O(3)-Sr(1)-O(4) | 119.65(14) |
|  |  | O(9)-Sr(1)-O(4) | 154.5(2) |
|  |  | O(2)-Sr(1)-O(4) | 57.09(11) |
|  |  | O(2)-Sr(1)-O(4) | 57.09(11) |
|  |  | O(5)-Sr(1)-O(4) | 56.55(15) |
|  |  | O(5)-Sr(1)-O(4) | 56.55(15) |
|  |  | O(3)-Sr(1)-O(7) | 65.80(15) |
|  |  | O(3)-Sr(1)-O(7) | 65.80(15) |
|  |  | O(9)-Sr(1)-O(7) | 124.7(2) |
|  |  | O(2)-Sr(1)-O(7) | 70.74(13) |
|  |  | O(2)-Sr(1)-O(7) | 70.74(13) |
|  |  | O(5)-Sr(1)-O(7) | 123.79(15) |
|  |  | O(5)-Sr(1)-O(7) | 123.79(15) |
|  |  | O(4)-Sr(1)-O(7) | 80.8(2) |
|  |  | O(3)-Sr(1)-O(9) | 115.17(15) |
|  |  | O(3)-Sr(1)-O(9) | 115.17(15) |
|  |  | O(9)-Sr(1)-O(9) | 56.9(3) |
|  |  | O(2)-Sr(1)-O(9) | 108.43(13) |
|  |  | O(2)-Sr(1)-O(9) | 108.43(13) |
|  |  | O(5)-Sr(1)-O(9) | 54.98(14) |
|  |  | O(5)-Sr(1)-O(9) | 54.98(14) |
|  |  | O(4)-Sr(1)-O(9) | 97.67(19) |
|  |  | O(7)-Sr(1)-O(9) | 178.43(19) |
|  |  | O(8)-Sr(2)-O(8) | 90.3(3) |
|  |  | O(8)-Sr(2)-O(10) | 129.83(15) |
|  |  | O(8)-Sr(2)-O(10) | 129.83(15) |
|  |  | O(8)-Sr(2)-O(5) | 150.55(19) |
|  |  | O(8)-Sr(2)-O(5) | 69.09(16) |
|  |  | O(10)-Sr(2)-O(5) | 79.21(14) |
|  |  | O(8)-Sr(2)-O(5) | 69.09(16)) |
|  |  | O(8)-Sr(2)-O(5) | 150.55(19) |
|  |  | O(10)-Sr(2)-O(5) | 79.21(14) |
|  |  | O(5)-Sr(2)-O(5) | 120.0(2) |
|  |  | O(8)-Sr(2)-O(6) | 69.98(16) |
|  |  | O(8)-Sr(2)-O(6) | 69.98(16) |
|  |  | O(10)-Sr(2)-O(6) | 143.8(3) |
|  |  | O(5)-Sr(2)-O(6) | 82.96(14) |
|  |  | O(5)-Sr(2)-O(6) | 82.96(14) |
|  |  | O(8)-Sr(2)-O(1) | 112.36(17) |
|  |  | O(8)-Sr(2)-O(1) | 112.36(17) |
|  |  | O(10)-Sr(2)-O(1) | 82.2(2) |
|  |  | O(5)-Sr(2)-O(1) | 61.01(11) |
|  |  | O(5)-Sr(2)-O(1) | 61.01(11) |
|  |  | O(6)-Sr(2)-O(1) | 61.7(2) |
|  |  | O(8)-Sr(2)-O(10) | 87.04(17) |
|  |  | O(8)-Sr(2)-O(10) | 87.04(17) |
|  |  | O(10)-Sr(2)-O(10) | 69.4(3) |
|  |  | O(5)-Sr(2)-O(10) | 111.59(13) |
|  |  | O(5)-Sr(2)-O(10) | 111.59(13) |
|  |  | O(8)-Sr(2)-O(5) | 150.55(19) |
|  |  | O(6)-Sr(2)-O(10) | 146.8(2) |
|  |  | O(1)-Sr(2)-O(10) | 151.6(2) |
|  |  | O(2)-Sr(3)-O(2) | 168.8(4) |
|  |  | O(2)-Sr(3)-O(6) | 103.4(2) |
|  |  | O(2)-Sr(3)-O(6) | 68.3(2) |
|  |  | O(2)-Sr(3)-O(6) | 68.3(2) |
|  |  | O(2)-Sr(3)-O(6) | 103.4(2) |
|  |  | O(6)-Sr(3)-O(6) | 88.4(3) |
|  |  | O(2)-Sr(3)-O(7) | 84.5(2) |
|  |  | O(2)-Sr(3)-O(7) | 104.0(2) |
|  |  | O(6)-Sr(3)-O(7) | 172.1(2) |
|  |  | O(6)-Sr(3)-O(7) | 95.15(17) |
|  |  | O(2)-Sr(3)-O(7) | 104.0(2) |
|  |  | O(2)-Sr(3)-O(7) | 84.5(2) |
|  |  | O(6)-Sr(3)-O(7) | 95.15(17) |
|  |  | O(6)-Sr(3)-O(7) | 172.1(2) |
|  |  | O(7)-Sr(3)-O(7) | 82.2(3) |
|  |  | O(2)-Sr(3)-O(8) | 58.68(15) |
|  |  | O(2)-Sr(3)-O(8) | 120.81(15) |
|  |  | O(6)-Sr(3)-O(8) | 65.91(19) |
|  |  | O(6)-Sr(3)-O(8) | 110.6(2) |
|  |  | O(7)-Sr(3)-O(8) | 119.0(2) |
|  |  | O(7)-Sr(3)-O(8) | 64.84(18) |
|  |  | O(2)-Sr(3)-O(8) | 120.81(15) |
|  |  | O(2)-Sr(3)-O(8) | 58.68(15) |
|  |  | O(6)-Sr(3)-O(8) | 110.6(2) |
|  |  | O(6)-Sr(3)-O(8) | 65.91(19) |
|  |  | O(7)-Sr(3)-O(8) | 64.84(18) |
|  |  | O(7)-Sr(3)-O(8) | 119.0(2) |
|  |  | O(8)-Sr(3)-O(8) | 175.5(3) |


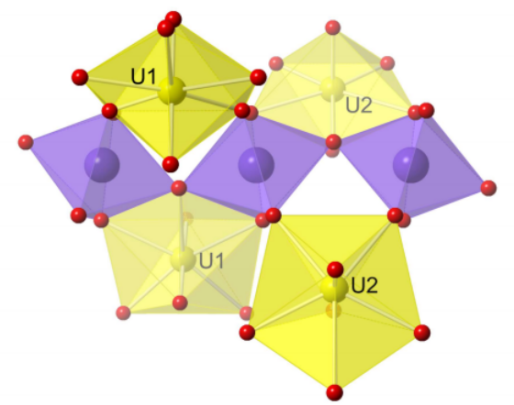


Figure S1**:** Local Te coordination and connectivity in Sr[(UO_2_)(TeO_5_)].
